# Graphene-Based Hybrid Fillers for Rubber Composites

**DOI:** 10.3390/molecules29051009

**Published:** 2024-02-26

**Authors:** Jian Wang, Shijiu Li, Li Yang, Baohua Liu, Songzhi Xie, Rui Qi, Yanhu Zhan, Hesheng Xia

**Affiliations:** 1 College of Food and Biological Engineering, Chengdu University, Chengdu 610106, China; wangjian@cdu.edu.cn (J.W.); 13398223058@163.com (S.L.); liubaohua@cdu.edu.cn (B.L.); xiesongzhi@cdu.edu.cn (S.X.); qirui@cdu.edu.cn (R.Q.); 2 Institute for Advanced Study, Chengdu University, Chengdu 610106, China; yanglipoly@cdu.edu.cn; 3 State Key Laboratory of Polymer Materials Engineering, Polymer Research Institute, Sichuan University, Chengdu 610065, China

**Keywords:** graphene, rubber, hybrid fillers, carbon black, silica, carbon nanotubes, metal oxide, nanowires, 3D network, enhancing mechanism

## Abstract

Graphene and its derivatives have been confirmed to be among the best fillers for rubber due to their excellent properties, such as high mechanical strength, improved interface interaction, and strain-induced crystallization capabilities. Graphene rubber materials can be widely used in tires, shoes, high-barrier conductive seals, electromagnetic shielding seals, shock absorbers, etc. In order to reduce the graphene loading and endow more desirable functions to rubber materials, graphene-based hybrid fillers are extensively employed, which can effectively enhance the performance of rubber composites. This review briefly summarizes the recent research on rubber composites with graphene-based hybrid fillers consisting of carbon black, silica, carbon nanotubes, metal oxide, and one-dimensional nanowires. The preparation methods, performance improvements, and applications of different graphene-based hybrid fillers/rubber composites have been investigated. This study also focuses on methods that can ensure the effectiveness of graphene hybrid fillers in reinforcing rubber composites. Furthermore, the enhanced mechanism of graphene- and graphene derivative-based hybrid fillers in rubber composites is investigated to provide a foundation for future studies.

## 1. Introduction

Rubber is an elastomer material that can sustain large reversible deformations and can be used in different products, such as tires, gloves, shoe soles, seals, insulators, and dampers [1,2,3,4]. The different types of rubber include natural rubber (NR), styrene–butadiene rubber (SBR), nitrile rubber (NBR), butyl rubber (BR), isobutylene isoprene rubber (IIR), ethylene-propylene-diene monomer (EPDM) rubber, and polydimethylsiloxane (PDMS)/silicone rubber (SR) [5,6]. They can be applied in transportation, construction, machinery, electronics, and energy sectors. With the development of modern industries, conventional rubber materials cannot satisfy the increasing demand for applications. Therefore, the development of high-performance multifunctional rubber composites is of great significance. In recent years, graphene and its derivatives have been extensively investigated as one kind of novel filler to improve the properties of rubber [7,8,9,10].

Owing to their large specific surface areas, high strengths, and high carrier mobilities, graphene, and its derivatives, exhibit high Young’s modulus, structural flexibilities, excellent thermal and electrical conductivities, and barrier properties [11,12,13,14,15,16,17]. Therefore, graphene and its derivatives, such as graphene oxide (GO), reduced GO (rGO), modified GO (mGO), graphene aerogel, and graphene foam, can effectively improve the properties of rubber materials. This in turn can facilitate the preparation of high-performance multifunctional rubber composites [7,18,19,20]. As a prospective filler in rubber materials, graphene and its derivatives possess several advantages: (1) The two-dimensional (2D) atomically thick carbon structure of graphene can result in an extraordinary modulus that is highly flexible and lightweight. Graphene can achieve a better mechanical reinforcement effect, even in small quantities, compared to other conventional fillers. (2) Owing to its high carrier mobility, graphene can enable rubber composites with high electrical and thermal conductivities, particularly by constructing segregated graphene networks in the rubber matrix. (3) The special structure of graphene can improve the gas barrier property. (4) Graphene and its derivatives have a high specific area, which can lead to more interfacial contacts, larger effects on molecular chains, and an enhanced strain-induced crystallization ability in comparison with other common rubber fillers. (5) In the case of GO, certain polar groups exist, such as epoxide, hydroxyl, and carboxyl. These groups render the modification of graphene easy, based on functionality molecules that can enhance the interaction between the filler and rubber matrix [2,8,21,22]. Thus, graphene and its derivatives have attracted widespread attention in recent years, resulting in a new technological revolution in the rubber industry [23,24,25].

However, compared to the widely used fillers in industrial production, including silica, carbon black (CB), and clay, the large-scale industrial production of graphene is extremely expensive. Rubber merchandise, particularly tires and shoe soles, should generally be filled with large amounts of CB or silica in practical applications. In certain cases, the content of fillers is even as high as 70% to ensure a balance between performance and cost. Therefore, completely replacing conventional and inexpensive fillers with expensive graphene is not a feasible option. A widely accepted strategy is to use a small amount of graphene (graphene derivatives) to partially replace conventional fillers, improve the properties of rubber composites, and reduce production costs. Moreover, the self-aggregation of graphene can be prevented, to a certain extent, following the addition of another filler, and a few shortcomings of graphene, such as magnetic properties and flexibility, can be compensated by other fillers, endowing rubber materials with more properties [26,27,28].

Therefore, analyzing the combination of graphene (and graphene derivatives) with other fillers to enhance rubber materials is essential. So, the research of graphene (and graphene derivative) hybrid fillers/rubber composites which include different preparation methods and enhanced properties, and the application of these composites, has been widely reported in recent years [8,29,30,31,32]. To ensure that graphene can exert the same enhancement and modification effects on the hybrid fillers as those observed when graphene is added individually, three issues must be addressed: (1) Graphene (graphene derivatives) and other fillers must be well dispersed in the rubber matrix. (2) A strong interaction should exist between the graphene (graphene derivatives)-based hybrid fillers and the rubber matrix. (3) The synergistic effect of graphene (graphene derivatives) and other fillers should be excellent. The methods that address these issues are very important for the research of graphene (graphene derivatives) hybrid fillers/rubber composites. Additionally, understanding the enhanced mechanisms of graphene (graphene derivatives) hybrid fillers in the rubber matrix is significant for deeper exploration in this field.

**Scheme 1:**
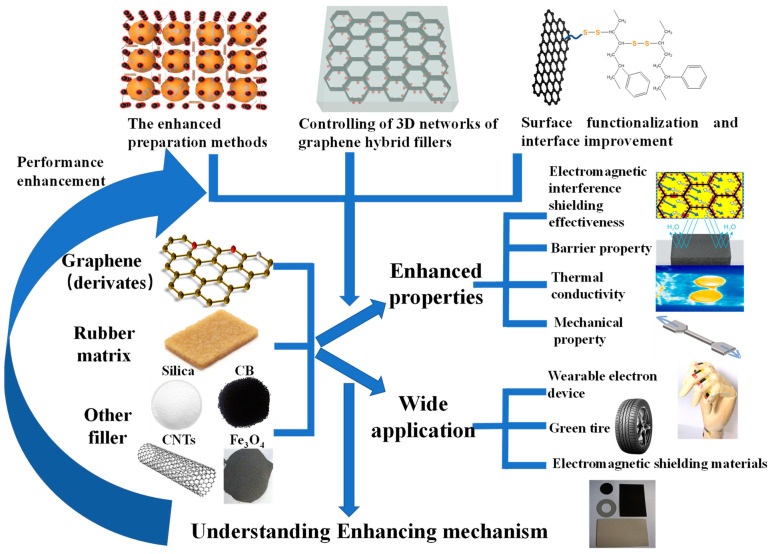
The primary context of this study.

In this review, different graphene hybrid fillers/rubber composites and graphene derivate-based hybrid fillers/rubber composites, such as graphene/silica, graphene/CB, graphene/CNT, graphene/metal oxide, and graphene/one-dimensional nanowires, are examined. It focuses on the preparation methods, different properties, and applications of graphene (graphene derivatives)-based hybrid fillers in rubber composites. Furthermore, three measures have been summarized, namely, enhanced preparation methods, con-trolling of 3D networks of graphene hybrid fillers, and surface functionalization and interface improvement, to address the aforementioned issues and improve the properties of rubber composites. Finally, the enhanced mechanisms of graphene hybrid fillers in a rubber matrix have been described, which can facilitate the improvement of the properties and the future development of graphene-based hybrid fillers in rubber composites. Scheme 1 illustrates the primary context of this study.

## 2. Rubber Composites with Different Graphene Hybrid Fillers

### 2.1. Graphene/CB

CB is a commonly used filler in the production of industrial rubber and is widely applied in the industrial preparation of tires and shoe soles. It is an indispensable component of rubber that enhances the performance of the rubber matrix and reduces costs. Therefore, rubber composites reinforced with graphene (graphene derivatives) and CB (GE/CB) hybrid fillers have been widely studied as rubber materials. The addition of graphene or graphene derivatives to a CB rubber matrix not only improves the thermal conductivity, mechanical properties, fatigue, and various properties of rubber composites but also expands their application scope [33,34,35,36,37,38,39,40,41,42,43,44,45,46,47,48,49,50,51,52,53,54,55].

Two common methods are used to prepare GE/CB hybrid rubber composites. The first is a solution mixed with graphene and CB, which is then mechanically mixed with a rubber matrix. The second method involves the preparation of a graphene/rubber masterbatch, which is then mechanically mixed with CB and a rubber matrix. Zhang et al. [34] prepared CB-rGO hybrid fillers using a solution mixing method. The fillers were mixed with SBR to manufacture the composites based on two-roll mixing and their performance was tested. They determined that CB could hinder the restacking of the rGO sheets. The tensile properties of the SBR/CB-rGO composites were significantly improved compared to those of the SBR/CB composites, and the volume resistivity was reduced. However, no difference was observed in the thermal stability between the pristine SBR and the CB- or CB/rGO-filled SBR composites. Yang et al. [35] prepared GO/NR and rGO/NR masterbatches. Subsequently, they were mixed with CB and rubber using a mechanical mixing method. The effect of different contents of GO or rGO was investigated as substitutes for CB in NR. The static and dynamic mechanical properties of the composites were significantly improved by the addition of small amounts of GO or rGO nanosheets. Particularly, higher flex-cracking resistance and lower heat build-up were achieved. Song et al. [46] prepared a masterbatch with low-defect graphene flakes and SBR, which was mixed with CB and other additives in a Banbury mixer. The graphene nanoplatelets (GNPs) improved the modulus, tensile strength, thermal and electrical conductivities, and gas barrier properties of the rubber composites. Guo et al. [42] prepared a GO/NR masterbatch, which was mechanically mixed with CB to obtain GO-CB/NR composites. The results indicated that the complex filler dispersion in the NR matrix improved because of the isolation effect among the different fillers. Furthermore, the strain-induced crystallization ability of the CB/NR was enhanced by the addition of GO, and the modulus at 100% strain and tear strength of the composites were also improved. In comparison with the CB/NR composites, the addition of GO significantly increased the fatigue lives of the composites. Particularly, the GO-CB/NR composites exhibited clear advantages in terms of fatigue resistance and durability.

Direct latex mixing is another method of preparing GE/CB hybrid filler rubber composites; this method is particularly used for composite films. Roy et al. [44] reported a novel method that intercalated CB into graphite (GN) layers using an ammonium sulfate electrolyte at a direct current voltage of 10 V to obtain CB-GN hybrid fillers. The hybrid fillers were then mixed with SBR latex to prepare CB-GN/SBR composite films. The composites exhibited a considerably high specific capacitance (244 F·g^−1^) and ferromagnetism within a small magnetic field. Additionally, the hybrid fillers increased the moduli and tensile strengths of the composites by 72% and 34%, respectively. These remarkable properties of the rubber nanocomposites paved the way for the application of polymeric materials in diverse electronics.

GE/CB hybrid filler rubber composites can also be used in sensors [33,56,57]. Cai et al. [33] fabricated hybrid fillers by mixing graphene nanosheets (referred to as GNPs in the study [33]) and CB in a naphtha solution, which was then mixed with SR via melt blending. The experimental results indicated that the electrical conductivity of the composite was the best when the mass ratio of GNPs to the CB filler was 4:2. Additionally, the percolation threshold of the CB/GNPs/SR composite was 0.18 vol% lower than that of the CB/SR composite. They also determined that the addition of GE increased the force range, and the linearity of GNPs/CB/SR was higher than that of the filler that contained only CB. The reproducibility of the GNPs/CB/SR composite was superior to that of the CB/SR composite. This result indicates that GNPs/CB/SR composites have high performance and sensitivity for use in flexible piezoresistive sensors. Song et al. [57] first modified CB with a silane coupling agent and then filled SR with the modified CB and different amounts of graphene to obtain SR/GE/conductive CB (CCB) composites. The CCB exhibited better dispersibility in the rubber matrix, and blending with graphene improved the sensing performance of the composite material. Specifically, the composite exhibited high strain sensitivity with gauge factors of 326 and 1.89 × 10^4^ at strain ranges of 8–10% and 28–44%, respectively. Furthermore, the composite exhibited adequate durability after 1000 cyclic strains. The composite could effectively detect human movements, such as pulse beats, swallowing, and muscle contraction, indicating its potential application in the field of wearable electronic devices.

Overall, GE/CB are the most widely studied hybrid fillers. The addition of GE and its derivatives can enhance the mechanical and thermal conductivity of CB/rubber composites, compensating for the shortcomings of single carbon black performance. A small amount of added GE can replace a significant amount of CB. Various GE/CB rubber products, such as tires and shoe soles, are used in practical rubber industries. Table 1 summarizes the preparation methods, improved properties, and applications of different graphene (graphene derivatives)/CB rubber composites, including the graphene hybrid filler type and rubber matrix type.

### 2.2. Graphene/Silica

Silica (SiO_2_) is an important filler in the modern rubber industry. In comparison with CB, the addition of silica can increase the anti-slippery performance and reduce the rolling resistance of rubber materials [60,61]. This makes rubber composites more suitable for the requirements of green production. Therefore, graphene (graphene derivatives)/silica hybrid filler-reinforced rubber has been widely studied in recent years [58,62,63,64,65,66,67,68,69,70,71,72,73,74,75,76].

Typically, a GE/rubber or GE/silica/rubber masterbatch is prepared, followed by mechanically mixing it with other agents and a rubber matrix; this is a common method used for preparing GE/silica hybrid rubber composites. Zhang et al. [62] studied GO and silica-reinforced SBR composites and focused on the effect of GO on their fatigue properties. The composites were fabricated by mechanically compounding a GO/SBR masterbatch with silica. The experimental results indicated that the GO sheets exhibited good dispersibility in the SBR matrix. The filler network composed of GO and silica improved after the addition of GO, thereby enhancing the mechanical properties of the composites. The dispersion of GO and silica was enhanced during the fatigue process, and the cross-linking density of the composites gradually decreased. The curves of stress vs number of cycles (S-N curves) indicated that the fatigue lifetime of the composites improved after the addition of GO. Furthermore, the strain energy density could be used as a predictor of fatigue life in the case of the GO/silica/SBR composites. Gu et al. [63] prepared a masterbatch of graphene nanosheets (referred to as GNs in the study [63])/NR, which was then mechanically mixed with SiO_2_, other NR materials, and other additives to obtain GNs/SiO_2_/NR composites. The results verified that GNs improved the mechanical properties and solvent resistance of the SiO_2_/NR composites. Moreover, the thermal conductivity and electrical conductivity of the 2.0 wt% GNs/SiO_2_/NR composites were increased by 60% and five orders of magnitude in comparison with the SiO_2_/NR composites, reaching 0.29 W·m^−1^·K^−1^ and 4.2 × 10^−8^ S/m, respectively. These results suggest that GNs can be suitable candidates for improving the thermal conductivity and antistatic properties of energy-saving tires.

Several researchers have also explored the electrostatic self-assembly or hydrogen bonding between graphene and silica as a new method for preparing graphene/silica/rubber composites. In this method, the modified silica and mGO were tightly combined by electrostatic interactions or hydrogen bonding to obtain a hybrid filler. Subsequently, the graphene/silica/rubber composites were prepared by mechanical mixing or the solution mixing method [3,77,78,79,80]. This method effectively increased the synergistic enhancement effect of the two fillers and prevented the re-aggregation of graphene. Liu et al. [72] prepared positively charged mesoporous silica, which exhibited strong electrostatic self-assembly with negatively charged GO. The special structure of mesoporous silica prevented graphene aggregation, and the addition of a mixed mesoporous silica/graphene filler to the SBR matrix effectively improved the thermal conductivity of the composite. When three parts of the mixed fillers were added, the thermal conductivity of the composite reached 0.424 W·m^−1^·K^−1^, which was approximately 183% of the thermal conductivity of the neat SBR. Cao et al. [80] also prepared γ-aminopropyltriethoxysilane (KH550)-modified GO (KGO) and Si69-modified SiO_2_ (MS) hybrid nanoparticles (MSKGO) via hydrogen bond self-assembly to reinforce the properties of NR. The composite of NR and MSKGO (NRMSKGO) was converted into a covalently bonded network structure through possible condensation and free-radical reactions. The addition of the MSKGO filler increased the storage modulus and energy-dissipation capability, whereas the loss factor and elongation at break decreased with the increasing GO content of the NRMSKGO composites; this was attributed to the enlarged interfacial area and the synergistic reinforcing effect of the covalently bonded MSKGO. Additionally, the MSKGO hybrid fillers increased the wear resistance, decreased the rolling resistance, and increased the wet-skid resistance, which facilitated the preparation of green tire composites.

The sol–gel method [64,66,81,82,83] and chemical reaction method [84,85,86,87] have also been considered for the preparation of graphene (graphene derivatives)/silica hybrid fillers mixed with a rubber matrix. In this way, it can also effectively improve the graphene/silica hybrid filler’s reinforcement of the rubber matrix. Charoenchai et al. [64] used the sol–gel method to prepare a silica–GO hybrid filler by using tetraethyl orthosilicate as the precursor of silica (SiO_2_). This method ensured that the silica was completely incorporated on the GO surface. Subsequently, the SiO_2_@GO hybrid fillers were mixed with NR using a two-roll mill to obtain the composites. The SiO_2_@GO induced cross-linking in the NR chains and increased the cross-linking density of the NR composites. Therefore, it reduced strain-induced crystallization under higher strains, which significantly increased the elongation at break and hardness of the composites.

**Figure 1:**
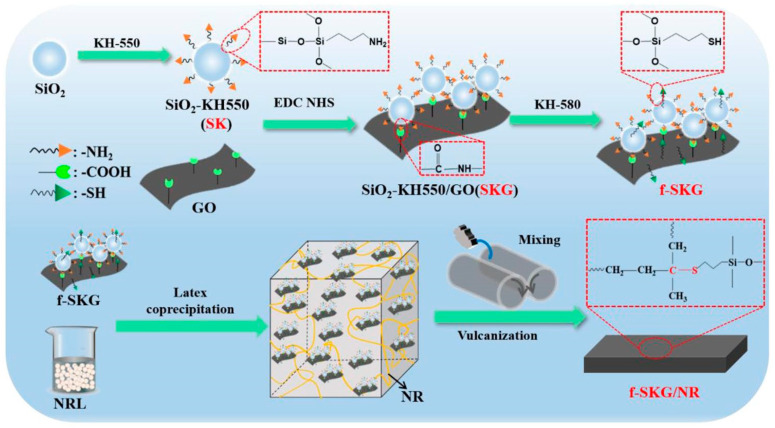
The fabrication procedure for f-SKG/NR composites (reprinted/adapted with permission from Ref. [84], 2024, Springer Nature).

Duan et al. [84] used KH550 to modify silica, which introduced the amino group on the surface of the silica before reacting with the carboxyl group on the surface of the GO; here, 1-ethyl-3-(3-dimethylaminopropyl) carbodiimide (EDC, 98.5%) and N-hydroxy succinimide (NHS) were used to obtain the f-SiO_2_-KH550/GO (f-SKG) hybrid fillers. The filler was then mixed with NR using the latex co-precipitation method. Additionally, the sulfhydryl functional groups on the surface of the f-SKG strengthened the interaction of the hybrid fillers with the NR matrix during vulcanization. Figure 1 illustrates the fabrication process of the composites. The good synergy between graphene and silica, and the enhanced filler–matrix interface interaction, endowed the NR composites with excellent mechanical properties, low heat-generation performance, and adequate thermal conductivity. This rendered them suitable for use in green tires. Liu et al. [85] introduced a new method, wherein GO and γ-(2,3-epoxypropoxypropyl) trimethoxysilane (KH560) modified silica reacted with each other based on covalent bonding with the help of triethylamine to obtain connected hybrid nanofillers. The hybrid fillers were then mixed with the NR latex to obtain NR composites. Covalent bridging has been proven to increase the interaction between the rubber and fillers, thereby enhancing the orientation of chain segments in the interfacial regions. Thus, with the addition of a small amount of GO/silica hybrid nanofillers, the tensile strength could be improved without decreasing the elongation at break of the composites.

However, the electrostatic self-assembly, sol–gel, and chemical reaction methods are not suitable for high-silica-content rubber systems, which are widely used in the modern rubber industry. The aggregation of silica occurs in high-silica systems, which may embed graphene in the aggregates and limit its enhancement effect. Wang et al. [65] used wet compounding and an in situ reduction latex mixing process to mix NR, rGO, and silica with a novel interface modifier, cystamine dihydrochloride (CDHC). This further enhanced the properties of the rGO/SiO_2_/NR composites with a high silica content (60 phr). Here, CDHC served as a coagulation agent through electrostatic interactions with rGO, SiO_2_, and latex rubber particles during the latex-based preparation process. Conversely, CDHC served as an interface modifier in the obtained rGO/SiO_2_/NR rubber composites. Furthermore, CDHC promoted vulcanization, which was confirmed by the bound rubber content and cross-link density. In comparison with the rGO/SiO_2_/NR composites prepared using the conventional masterbatch mechanical mixing method, the dispersion of both rGO and SiO_2_ was improved in the composites prepared by wet compounding and the in situ reduction latex mixing process. The rubber composites prepared using this new method exhibited excellent mechanical properties and low water vapor permeability. Moreover, the DMA test suggested that the tan(δ) values of the composites at 60 °C decreased with the increasing graphene content at a low strain; however, the values increased at a higher strain, providing a unique advantage for this material in rubber tire applications.

Paraffin@SiO_2_ microcapsules have also been mixed with graphene (graphene derivatives) and rubber to prepare phase change materials with enhanced thermal conductivity. This special structure renders the composites better suited for passive thermal regulation and energy storage applications. Song et al. [88] used paraffin@SiO_2_ microcapsules (Pn@SiO_2_) as the filler, which were mixed with graphene and SR to obtain shape-stabilized phase change materials via solution mixing and blending. Owing to their special structures, the graphene and Pn@SiO_2_ were completely encapsulated in the SR matrix. The composites exhibited excellent mechanical properties, thermal conductivities, and low leakage rates. They also exhibited different thermal energy storage capacities when different amounts of microcapsules were added to the composites, this improved their application in the field of thermal energy storage. Kang et al. [89] prepared paraffin@SiO_2_/GE/SR composites via mechanical mixing using a planetary centrifugal mixer. The addition of silica and graphene significantly increased the thermal conductivity of the composites, whereas the SiO_2_ shell and SR skeleton restricted the leakage of liquid Pa. These excellent properties render these composites better suited for passive thermal regulation applications.

Silica, a commonly used reinforced filler in the rubber industry, finds extensive applications in the field of green tires. The addition of graphene (graphene derivatives) can significantly enhance the mechanical properties, rolling resistance, and wet slip resistance of rubber composites. The common method involves the self-assembly of graphene (graphene derivatives) with silica to reinforce the rubber, thereby greatly improving several properties of the GE/SiO_2_/rubber composites. However, green tire rubber composites often require a very high silica content, and further research is needed to understand the reinforcing effects of graphene in rubber matrices with high silica content. Table 2 summarizes the preparation methods, improved properties, and applications of different graphene (graphene derivatives)/silica rubber composites, including the graphene hybrid filler type and rubber matrix type.

### 2.3. Graphene/CNTs

CNTs have a perfect hexagonal structure with superior mechanical, electrical, and chemical properties and are commonly used as one-dimensional (1D) reinforcing fillers. Therefore, CNTs can be used to improve the properties of rubber and expand its potential applications [29,30,94,95]. In addition, introducing hybrids of 1D CNTs and 2D graphene with similar properties can prevent the aggregation of graphene and promote strong interfacial interactions with the rubber matrix. Therefore, graphene (graphene derivatives)/CNTs hybrid fillers can synergistically improve rubber materials compared to the effectiveness of these fillers individually. Moreover, the blending of graphene and CNTs can generate 1D and 2D hybrid bilayer conductive networks in the rubber matrix more effectively, which can further enhance the electrical conductivity and electromagnetic shielding performance of composite materials. Thus, graphene (graphene derivatives)/CNTs/rubber composites can be efficiently used in sensors and other electrical devices [28,96,97,98,99,100,101,102,103,104,105,106,107,108].

Typically, the solution mixing method with tetrahydrofuran (THF) and hexane is used for preparing GE/CNT hybrid rubber composites [103,109,110,111,112]. Yang et al. [103] used the solution mixing method with self-assembled CNTs and graphene (referred to as GR in the study [103]) to prepare the CNTs/GR/SR vinyl methyl silicone (VMQ) composites in THF. The CNTs were treated with p-octyl polyethylene glycol phenyl ether (OP-10)/cetyltrimethylammonium bromide (CTAB), whereas the GR was treated with sodium dodecyl sulfate (SDS). The electrostatic bonds between graphene and CNTs were enabled owing to the presence of CTAB, OP-10, and SDS. The addition of GR improved the dispersion of the CNTs, and the composites exhibited a significantly low percolation threshold of 0.92 wt%, which was 53% lower than that of the CNTs/VMQ composites. Additionally, the self-assembled CNTs-GR/VMQ composites exhibited a monotonic and more stable resistance response under cyclic loading with no significant downward drift or shoulder peak. Oh et al. [111] used the solution mixing method to prepare the CNT/graphene hybrid fillers/PDMS composites. The CNT/graphene hybrid system exhibited synergistic effects that prevented agglomeration of CNTs and graphene restacking with reduced contact resistance owing to the formation of 1D (CNT)–2D (graphene) interconnection. Consequently, the hybrid fillers/PDMS had the highest electrical conductivity compared to those of the composites with only one CNT or graphene filler. Additionally, hybrid filler nanocomposites that contained a low fraction of conductive fillers retained their electrical conductivity in the range of 10^−5^–10^−4^ S/cm and 10^−3^−10^−2^ S/cm at 0.4 and 0.6 wt%, respectively, when strained up to 60%; this demonstrated their potential for applications as stretchable conductors. Kong et al. [113] prepared rGO and a catalyst precursor at high temperatures and then used acetone as a carbon source to uniformly grow CNTs on the surface of rGO. These hybrid fillers produced a lower interfacial contact electrical resistance and were well-dispersed in the PDMS matrix to form a ternary hierarchical architecture. This endowed the composites with a low reflection coefficient and wide effective absorption bandwidth, significantly enhancing the electromagnetic absorption capacity of PDMS.

The latex mixing [100,101,114] and mechanical mixing [115,116,117] methods have also been used for preparing GE/CNT/rubber composites. Li et al. [100] combined GE and CNTs hybrid fillers to reinforce NR, wherein GE/CNT/NR composites were prepared using in situ reduction with NR latex. The high-energy dissipation effect of the hybrid fillers’ structure improved the fracture toughness and tensile strength of the composites while suppressing crack propagation in the GE/CNT/NR nanocomposites. Furthermore, they compared the GE/CNT hybrid fillers with other fillers, and the results indicated that the GE/CNT hybrid fillers were the best hybrid fillers for effectively enhancing the fracture toughness of rubber materials. Araby et al. [101] prepared multi-walled CNTs (MWCNTs)/GNP/EPDM rubber composites using the direct mechanical mixing method. The MWCNTs and GNPs combined to form a conductive network; here, the MWCNTs acted as long nanomaterials for electron and stress transport, whereas the GNPs were used as conduits to form locally conductive interconnects. The results indicated that the permeation threshold of the three-phase composite was 2.3 vol%, which was 88% lower than that of the two-phase composite. Furthermore, the three-phase composite exhibited superior mechanical properties, including tensile strength, Young’s modulus, and tear strength.

Kim et al. [116] prepared conductive dry adhesives (CDA) by mixing 1D CNTs and 2D carbon materials (CB, nano graphite, and graphene nanopowder) into conductive elastomer PDMS using the direct mechanical mixing method in a planetary mixer. The 1D−2D carbon hybrid fillers system were introduced to enhance the electrical percolation and maximize the conductivity in the elastomer matrix at low concentration (approximately 1 wt%) of fillers, with the optimized mixing ratio of CNTs and graphene set to 9:1. The composite film with optimized conditions exhibited superior stretchability over nearly 100% and electrical conductivity (approximately 100 Ω·cm) that was sufficient to operate light-emitting diodes. Owing to its excellent properties, this innovative CDA can be used in electrocardiogram monitoring systems as a cost-effective and reusable all-in-one-type electrode. Maya et al. [117] prepared rGO/CNT/chloroprene rubber (CR) composites via the direct mechanical mixing method. The non-covalent π–π interaction between CNTs and rGO and the secondary interaction between rGO/CNT and CR effectively resulted in a load transfer between the fillers and the rubber matrix. Consequently, composites were endowed with high thermal conductivities, low dielectric losses, and high energy storage efficiencies.

In summary, the GE/CNTs hybrid fillers can effectively enhance the electrical conductivity, EMI shielding, and various properties of rubber composites, making them suitable for potential applications in the field of sensors. However, a simple mixture of one-dimensional GE and two-dimensional CNTs may not be the optimal choice for effectively improving these properties. The construction of specific structures can more efficiently enhance these properties, a topic we will explore later. Table 3 summarizes the preparation methods, improved properties, and applications of different graphene (graphene derivatives)/carbon nanotube composites, including the graphene hybrid filler type and rubber matrix type.

### 2.4. Graphene/Metal Oxide

Metal oxides, such as Fe_3_O_4_, Al_2_O_3_, and ZnO, are commonly added fillers in the modern rubber industry. Various metal oxide/graphene/rubber composites have been widely studied in recent research.

Electromagnetic interference (EMI) pollution and microwave pollution are known to severely affect the operation and malfunction of certain sensitive electronic devices and negatively impact human health [122,123,124]. Therefore, the preparation of rubber composites with high-performance microwave absorption and EMI shielding is critical. Fe_3_O_4_ is a type of magnetic nanoparticle with adequate biocompatibility and low toxicity. These particles are often added to polymer matrices to enhance their microwave absorption and EMI-shielding properties [32,125,126]. Although several studies have reported that graphene can reinforce the EMI-shielding properties of rubber composites [127,128], the properties of these rubber composites have significant scope for improvement owing to the monotonous dielectric loss mechanism. Mixing graphene (graphene derivatives) and the magnetic Fe_3_O_4_ is considered an appropriate approach to achieve this improvement. Combining Fe_3_O_4_ with graphene can prevent the self-aggregation of graphene and endow the composites with excellent electrical, mechanical, EMI-shielding, and microwave-absorption properties [26,27,125,129,130,131,132,133,134].

Essabir et al. [135] added graphene oxide nanosheets (GONs) and ferromagnetic nanoparticles (Fe_3_O_4_) as hybrid fillers to hybrid nanocomposites based on SBR and polyvinylidene difluoride (PVDF). Masterbatches of PVDF/5 wt% nanoparticles (GONs and Fe_3_O_4_) were prepared by solution mixing in dimethylformamide, followed by mechanical mixing with the matrix (PVDF/SBR blend) to obtain the final composites. Owing to the synergy between the two nanofillers, Young’s modulus of the nanocomposite containing 5 wt% of the hybrid nanofiller was significantly improved (87%), whereas the strain at yield remained constant. Additionally, the degradation temperature of the rubber shifted from 464 to 472 °C with the addition of the hybrid fillers GON: Fe_3_O_4_ in the ratio of 2.5:2.5. Finally, the hybrid reinforcement exhibited a positive effect on the electrical and magnetic properties of the nanocomposites. The magnetic stress increased linearly and reached a maximum of 870 Pa when the hybrid filler content reached 5 wt%. This resulted in composites with excellent properties, such as EMI shielding and radar/infrared blocking.

To obtain a better enhancement effect, Fe_3_O_4_ should be attached to the graphene surface. Hu et al. [133] prepared rGO@Fe_3_O_4_ nanocomposites, which were developed using a facile thermal decomposition method. The Fe_3_O_4_ nanoparticles were better fixed on the rGO sheets via an in situ growth process. Subsequently, the rGO@Fe_3_O_4_ fillers were mechanically mixed with SR to obtain the rGO@Fe_3_O_4_/SR composites. The rGO@Fe_3_O_4_ filler endowed the composites with magnetic properties and provided a heterogeneous interface, leading to improved impedance matching, enhanced magnetic and interfacial polarization losses, and high thermal conductivity. Therefore, the rGO@Fe_3_O_4_/SR composites exhibited a high microwave-absorption efficiency and a wide absorption bandwidth. The minimum reflection loss value reached −59.4 dB at a frequency of 8.0 GHz, and the absorption bandwidth was 4.2 GHz for a thin layer with a thickness of 1.2 mm. Owing to their improved properties, the developed rGO@Fe_3_O_4_/SR composites can be potential candidates for use as high-performance microwave-absorbing materials in aerospace and flexible electronics.

**Figure 2:**
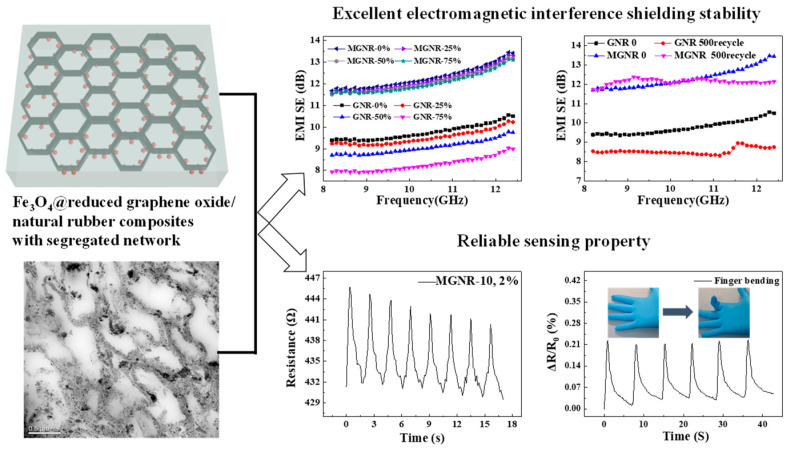
The rGO/Fe_3_O_4_/NR (MGNR) composites with a segregated network with better electromagnetic interference (EMI) shielding, EMI-shielding stability, and sensing properties (reprinted/adapted from Ref. [27], 2024, MDPI).

The fabrication of rubber composites with segregated network structures can increase the conductivity of the rubber material by several orders of magnitude, thereby improving the EMI-shielding performance of the composites [26,27,136]. Zhan et al. [26] and Wang et al. [27] successfully prepared rGO/Fe_3_O_4_/NR composites with segregated networks. GO/Fe_3_O_4_ hybrid fillers were prepared using electrostatic self-assembly. Subsequently, rGO/Fe_3_O_4_ with a segregated network was fabricated by latex mixing and in situ reduction. Fe_3_O_4_ nanoparticles decorated on the rGO sheets endowed the rGO/Fe_3_O_4_/NR composite with better magnetic properties than the rGO/NR composite with the same rGO content. The excellent magnetic properties, perfectly segregated network structure (Figure 2), and adequate electrical conductivity of the rGO/Fe_3_O_4_/NR composites improved the EMI-shielding properties of the composites. With the same rGO content of 10 phr, the EMI-shielding value of the rGO/Fe_3_O_4_/NR composite was 1.33 times higher than that of the rGO/NR composite. Furthermore, the presence of Fe_3_O_4_ nanoparticles endowed the rGO/Fe_3_O_4_/NR composite with excellent EMI-shielding stability under the conditions of different tensile strains, tensile permanent deformation, cyclic stretching, and cyclic bending; the EMI-shielding value was reduced by nearly 2.9%. This property is crucial for high-performance EMI-shielding materials. However, the EMI-shielding performance of the rGO/Fe_3_O_4_/NR composites decreased to a certain extent after the same treatment. Additionally, the rGO/Fe_3_O_4_/NR composite exhibited excellent sensing performance and stably monitored current signal changes even under cyclic stretching at extremely low strains (0.05%). Resistance signal changes were also stably and repeatedly monitored by the rGO/Fe_3_O_4_/NR composites when they were used to detect different human motions, such as wrist bending, speaking, finger bending, and blinking. This indicated that the composites can be used in flexible and wearable electronic devices in the future.

In addition to EMI-shielding and microwave-absorption applications, graphene/Fe_3_O_4_ hybrid fillers can be used to enhance the surface wettability and corrosion resistance of rubber composites.

Zhang et al. [129] prepared graphene Fe_3_O_4_ hybrid fillers using triiron tetroxide (Fe_3_O_4_) as a carrier and then mixed them with NBR using the solution mixing method. The hybridization of graphene and Fe_3_O_4_ improved the dispersion of graphene in the NBR matrix, resulting in interfacial interactions between the graphene and NBR, and endowing the composite material with excellent properties. When 4 phr of graphene/Fe_3_O_4_ hybrid fillers were added to the NBR matrix, the tensile strength of the composite was increased by approximately 90%. The addition of graphene and Fe_3_O_4_ endowed NBR with other properties, such as surface wettability and magnetic properties, which can provide useful guidance for the preparation of functional graphene/rubber composites.

Wang et al. [132] prepared different Fe_3_O_4_@carbon nanomaterials/NR nanocomposite coatings using latex mixing. Carbon nanomaterials, such as rGO, GO, and CNTs, were efficiently dispersed in the NR latex with the help of Fe_3_O_4_ nanoparticles. The barrier and anti-corrosion properties of various Fe_3_O_4_@carbon nanomaterials/NR nanocomposite coatings were compared using electrochemical impedance spectroscopy, optical microscopy, and electrical polarization curve studies. The experimental results indicated that the addition of carbon materials effectively improved the corrosion resistance and reduced the corrosion rate of rubber composites when compared to pure NR. Moreover, the nanocomposite coating exhibited highly flexible anticorrosive properties, and the corrosion rate (mm/y) was maintained at 5.2 × 10^−4^ mm/y after over 10 cyclic bends.

Apart from Fe_3_O_4_, several other metal oxides can be used as common additives or enhancement fillers for rubber. Therefore, studies have focused on graphene (graphene derivatives)/metal-oxide hybrid filler-enhanced rubbers.

**Figure 3:**
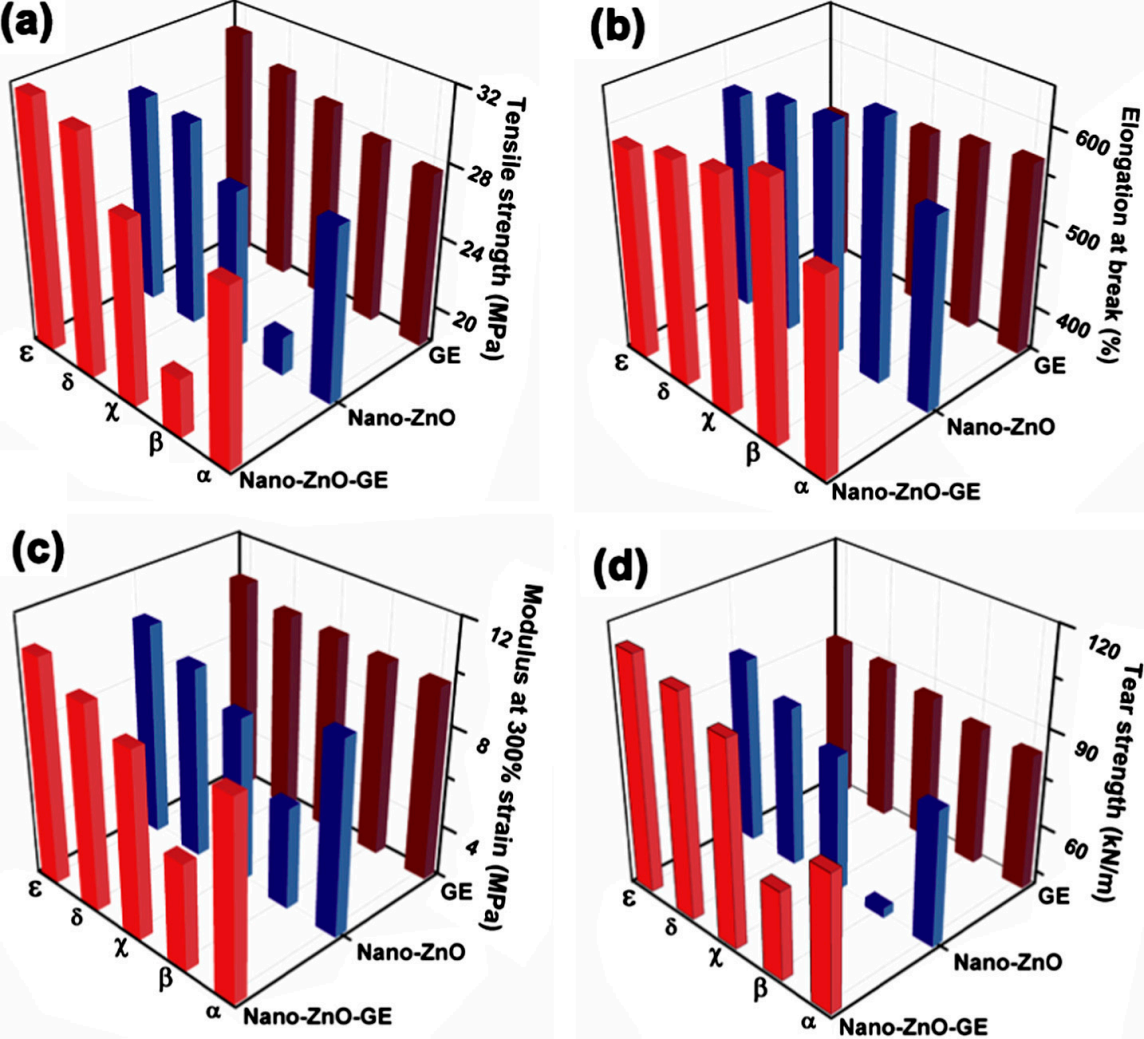
Comparison of mechanical properties of NR/Nano-ZnO–GE, NR/Nano-ZnO, and NR/GE nanocomposites. (**a**) Tensile strength; (**b**) elongation at break; (**c**) modulus at 300% strain; (**d**) tear strength. The symbol a represents 5 phr for conventional-ZnO, and b, v, d, and e represent 0.5, 1.0, 1.5, and 2.0 phr for nano-ZnO–GE, or 0.4225, 0.8450, 1.2675, and 1.6900 phr for nano-ZnO, or 0.0775, 0.1550, 0.2325, and 0.3100 phr for GE, respectively (reprinted/adapted with permission from Ref. [138], 2024, Elsevier).

Zinc oxide (ZnO), a vulcanization additive commonly used in rubber, serves as an essential cure activator for rubber cross-linking to obtain reliable cross-linking networks in rubber composites. Hence, ZnO is widely mixed with graphene to enhance the properties of rubber composites [137,138,139,140]. Lin et al. [138] fabricated ZnO-doped graphene (nano-ZnO-GE) using the thermal treatment and sol–gel method. They were mixed with NR to obtain composites via twin-roll milling. The presence of nano-ZnO on the GE sheets suppressed the aggregation of GE, acting as an efficient cure activator in the vulcanization process. This resulted in the formation of an excellent cross-linked network in the rubber matrix. In comparison with the NR containing 5 phr of ZnO, the nano-ZnO-GE-modified NR exhibited remarkably enhanced dynamic mechanical and static properties. Furthermore, the NR composite containing 1.5 phr of nano-ZnO-GE exhibited a tensile strength of approximately 31 MPa and a tear strength of nearly 109 kN/m, which were increased by 12.7 and 32.3%, respectively, compared to those of the pristine NR (Figure 3). Furthermore, the wet-grip property of nanocomposites was enhanced by 5.0%, and rolling resistance was reduced by 6.2%. Thus, nano-ZnO-GE significantly improves several properties of NR, rendering it more suitable for the preparation of green tires. Lin et al. [139] prepared graphene nanosheets (GNSs) decorated with ZnO nanoparticles (NZG) using a facile solvothermal method. As a substitute for conventional ZnO, NZG was mixed with the NR matrix using the mechanical mixing method. The results indicated that a low NZG content generated a higher vulcanization efficiency and a substantially stronger reinforcement effect on the mechanical performance and gas barrier properties of NR nanocomposites compared to GE, nano-ZnO, and 5 phr conventional ZnO. Furthermore, the NZG hybrid fillers increased the interfacial interaction between the filler and rubber matrix. Therefore, NZG can be a suitable substitute for the 5 phr conventional ZnO when applied to rubber composites. Xu et al. [137] prepared GO-supported zinc oxide (ZG) hybrids using electrostatic adsorption and in situ growth. The ZG hybrids were then introduced into chloroprene rubber (CR) via traditional two-roll mill mixing. ZG hybrids were used in the CR matrix to improve the properties of the rubber composites. The ZG hybrids were well-dispersed in the CR matrix, resulting in strong interfacial interactions with the CR molecules due to H and metal coordination bonding. This led to improved cross-linking efficiency and density of the composites, as well as excellent reinforcement effects, including increased tensile modulus at 300% elongation, tensile strength, and reinforcing index. Overall, this study provides valuable insights into the design of multifunctional rubber additives and the fabrication of high-performance rubber composites.

Some metal oxides, such as aluminum oxide (Al_2_O_3_), are often used as thermally conductive fillers to enhance the thermal conductivity of rubber materials. Additionally, blending metal oxides and graphene can achieve a better enhancement effect by reducing the amounts of metal oxides. Zhang et al. [141] used mechanical mixing to incorporate Al_2_O_3_ and GNSs into an SR matrix. The Al_2_O_3_ and GNSs formed a 3D thermal conductivity network with a high packing density in the SR matrix. This unique 3D network improved the dispersion of fillers in the rubber matrix and reduced the thermal resistance between the filler and matrix. Therefore, the thermal conductivity of the composite was effectively improved. The highest thermal conductivity that was achieved was 3.37 W·m^−1^·K^−1^, which was 47.1% higher than that of the composites with the same single filler. Li et al. [142] developed a novel 3D interconnected rGO@Al_2_O_3_ hybrid filler network using a GO-assisted gelation method. These fillers were mixed with NR by latex mixing to prepare the 3D rGO@Al_2_O_3_-NR nanocomposites. The 3D rGO@Al_2_O_3_ hybrid fillers were efficient heat transfer paths in NR, significantly enhancing several properties of the rubber composites. With a low filler loading of 18.0 vol%, the composites exhibited a considerably increased tensile strength (25.6 MPa) and a high thermal conductivity (0.514 W/(m·K)). Moreover, the filler network tended to orient under compression, resulting in an in-plane thermal conductivity of 3.233 W/(m·K) at 33.9 vol% filler content. Furthermore, the electrical resistance could be easily controlled by changing the proportion of Al_2_O_3_ coverage. This study presented new evidence that 3D rGO@Al_2_O_3_ hybrid fillers can be applied as advanced thermal management materials. Zhuang et al. [143] prepared novel core-shell structured fillers (F-GA) by covalently linking Al_2_O_3_ modified with poly (dopamine) and GO. Subsequently, F-GA/NR composites were fabricated based on vacuum-assisted filtration. The interfacial thermal resistance of the F-GA/NR composites was significantly reduced by the formation of covalent bonds and a 3D layered structure. Therefore, this structure endowed the composites with an excellent thermal conductivity of 0.863 W/m·K^−1^ at the F-GA filler content of 25 wt%, which was approximately five-fold higher than that of the pure NR. Additionally, the F-GA/NR composites exhibited improved mechanical properties and excellent electrical insulation, rendering them suitable for use in electronic packaging materials.

Other metal oxides, such as CeO_2_ and TiO_2_, were also combined with graphene to improve rubber properties [144,145]. Han et al. [144] prepared CeO_2_/graphene/phenyl silicone rubber (PMVQ) composites using the solution mixing method and focused on the thermal stability mechanism of the composites. The results of thermogravimetric analysis verified that CeO_2_ and graphene collectively improved the thermal stability of the PMVQ composites. Furthermore, the swelling test indicated that the aging of rubber composites was dominated by oxidation cross-linking at 300 °C for 48 h. Finally, the mechanical properties of the SR composites were significantly improved by the addition of CeO_2_ and graphene. The tensile strength of PMVQ composites containing 0.8 phr graphene and 2 phr CeO_2_ was 4.67 MPa, which was increased by 82.4% in comparison with those of PMVQ composites without graphene and CeO_2_. After aging, the elongation at break of PMVQ composites containing 0.8 phr graphene and 2 phr CeO_2_ remained at 180%; in PMVQ composites containing 1.5 phr graphene and 2 phr CeO_2_, the elongation at break remained at 213%. Kumar et al. [145] added few-layer graphene (FLG) and Fe_3_O_4_ or TiO_2_ as fillers to a room-temperature-vulcanized (RTV) SR matrix. The composites were prepared by mixing RTV-SR with nanofillers and vulcanizing at room temperature for 24 h. These results suggested that mechanical properties and magnetic stress relaxation could be improved by the addition of FLG/TiO_2_ fillers. Furthermore, the actuation displacements were the highest for RTV-SR/FLG-TiO_2_ at all voltages.

In summary, various metal oxides impart distinct properties to rubber composites. For instance, Fe_3_O_4_ enhances magnetic and electromagnetic shielding properties, ZnO improves vulcanization performance, and Al_2_O_3_ enhances thermal conductivity. When blended with graphene (GE), these metal oxides compensate for any shortcomings in GE performance, thereby broadening the application of rubber composites. Table 4 summarizes the preparation methods, improved properties, and applications of different graphene (graphene derivatives)/metal oxide composites, including the graphene hybrid filler type and rubber matrix type.

### 2.5. Graphene/1D Nanowires (Microwires, Nanofibers)

Nanowires (microwires, nanofibers), including silver nanowires, silicon carbide nanowires, cellulose nanofibers, and aramid nanofibers, are another type of 1D filler with high aspect ratios, continuous structures, low contact resistance, high strength, and superior conductivity [149,150,151,152]. Combining graphene and nanowires/microwires can solve the structural defects of graphene, reduce the contact resistance, and inhibit the agglomeration of the graphene (graphene derivatives) single filler, further improving the mechanical properties, electrical conductivity, thermal conductivity, EMI-shielding properties, and microwave absorption of rubber composites [153,154,155,156,157,158,159,160,161,162,163].

Enhancement of the EMI-shielding and microwave-absorption properties of rubber composites has been a major concern in recent research. Xu et al. [161] reported a simple shielding system that incorporated magnetic microwires (Co_60_Fe_15_Si10B15 glass-coated microwires) and graphene fibers into SR with various filler arrangements. The magnetic microwires and graphene fibers were incorporated into the SYLGARD(R) 184 silicone elastomer kit (Dow Corning), consisting of a base and curing agent, in a periodic and random manner. In comparison with periodic arrays, including continuous fillers, randomly dispersed arrays composed of short microwires/graphene fibers exhibited poor shielding effectiveness (SE) owing to low polarization and low aspect ratio effects, which were induced by the differences in conductivity and permittivity between the two regions. However, the highest shielding of 18 dB was provided by the MMMGGG periodic array with only 0.059 wt% filler loading, wherein the optimized arrangement enabled efficient wave attenuation based on the dielectric and magnetic properties of the microwires. Among the randomly dispersed arrays, the highest EMI SE value of 6 dB was achieved by the MþG composites because of polarization effects. Thus, the addition of hybrid fillers to periodic arrays can result in rubber composites with a high shielding efficiency and low microwave reflectivity despite the low filler loading, which can be used in various microwave applications. Wang et al. [154] prepared flexible and conductive NBR composite films with rGO and cellulose nanofibrils (CNF) by Pickering emulsion mixing using in situ reduction and hot-pressing technology. The addition of CNF assisted GO in the formation of a stable oil-in-water (O/W) Pickering emulsion gel. The rGO sheets specifically covered the closely stacked NBR microdroplets and formed a three-dimensional (3D) cross-linked conductive network, whereas the NBR microdroplets were isolated in the network. The special isolated NBR structure played a primary role in achieving the maximum high conductivity of 99 S·m^−1^, which was more than one order of magnitude greater compared to that of the composite materials prepared using traditional solution mixing. The 3D network structure of the rGO/CNF hybrid filler NBR film, with excellent flexibility and structural strength, exhibited a stable EMI SE of 25.81 dB in the X-band, and a decrease of 2.51% was observed in the EMI-shielding efficacy after 5000 cycles. Therefore, this composite can be used in wearable and portable medical equipment and electronic devices. Jeevakumari et al. [156] reported a direct mechanical mixing (two-roll mill) method for preparing flexible high-microwave-shielding NR composites by adding GNPs and cobalt nanowires. The experimental results verified that the addition of cobalt nanowires and GNP of 6 phr to NR offered the highest tensile and tear strengths of 30 MPa and 35 N/mm, respectively, with an improved micro-hardness of 64 Shore-A. Furthermore, the magnetic properties of the NR composite with graphene and cobalt nanowires exhibited a maximum magnetization and remanence of 940 and 610 emu, respectively. Additionally, the microwave shielding behavior of the composite reached its maximum compared to other composites, as it consisted of GNPs and cobalt nanowires of 6 phr. The maximum attenuation of 38.01 dB was observed at 18 GHz (J-band) microwave frequency. Therefore, the inclusion of magnetic cobalt nanowires significantly contributed to the dielectric, magnetic, and microwave attenuation behaviors of the composite, in comparison with other composites that contained only conductive particles. These flexible rubber composites can be used as shielding materials for electronic gadgets, radars, telecommunication cables, medical imaging, drone surveillance, automobiles, digital recordings, and microwave-shielding helmets.

With the assistance of microwire and graphene, the thermal conductivities of the composites can be significantly improved. Song et al. [155] used an ice-templated assembly strategy to construct silicon carbide (SiC) nanowires, rGO, and vertically aligned cellulose nanofiber filler networks via solution mixing and freeze-drying. The hybrid fillers were then infiltrated into poly (ethylene glycol)-grafted SR to prepare SiC nanowire (NW)/rGO/SR composites. The thermal conductivity of the composites increased with the increase in SiCNW and rGO contents. Particularly, the SiCNW/rGO/SR composite at the filler network content of 1.84 vol% exhibited a high thermal conductivity of 2.74 W/(m·K). The synergistic effect of the rGO and SiCNWs, macroscopically and microcosmically ordered filler network, and interface modification were responsible for the high thermal conductivity of the SiCNW/rGO/SR composites. This was critical for preparing rubber composites with high thermal conductivity and low filler content.

Hybrid-filler rubber composites can also be used for high-performance tire treads. Jung et al. [157] prepared an aramid nanofiber (ANF)/GOs hybrid filler by adding functionalized GOs (fGOs) to functionalized ANFs (fANFs) using a silane coupling agent. The fANF/fGO/SBR composites were then prepared using a Brabender Plasti-Corder internal mixer. In comparison with the fANF/SBR and fGO/SBR composites, the fANF/fGO/SBR composites exhibited improved mechanical properties. Particularly, the fANF/GO-reinforced rubber samples displayed an improvement of 18.2% with respect to abrasion resistance when one part of the fANF/GO hybrid filler was added. Furthermore, DMA testing results indicated that the rolling resistance predicted by tan(δ) at 60 °C was improved in the fANF-, fGO-, and fANF/GO-reinforced rubber compounds, while maintaining the wet-skid resistance. Remarkably, one part of the fANF/GO-reinforced rubber specimens exhibited an improvement of 21.8% in rolling resistance. These results suggest that the fANF/GO hybrid fillers can improve several properties of SBR composites and can be used as a high-performance green tire material.

In the end, combining graphene with a one-dimensional filler more effectively enhances the thermal conductivity, electrical conductivity, and mechanical properties of rubber composites. The synergistic effect of two-dimensional graphene and the one-dimensional filler is key to improving the performance of rubber composites. Table 5 summarizes the preparation methods, improved properties, and applications of graphene (graphene derivatives)/nanowires (microwire, nanofiber) composites, including the graphene hybrid filler type and rubber matrix type.

### 2.6. Other Binary Graphene-Based Hybrid Fillers

Apart from the aforementioned common fillers, certain other fillers mixed with graphene (graphene derivatives) to improve the properties of rubber have been reported by researchers.

Owing to its excellent magnetic properties, iron cobalt (FeCo) has been blended with graphene to improve the microwave-absorption properties of rubber composites [161,167]. Li et al. [167] mixed FeCo with graphene nanosheets (referred to as GS in the study [167]) via electrostatic interactions in solution to prepare FeCo/GS hybrid fillers. Subsequently, the hybrid fillers were mixed with SiC and natural latex to prepare microwave-absorbing composites with a thickness of 2 mm. The results indicated that the addition of GS or FeCo/GS hybrid fillers to the SiC/NR samples significantly increased the reflection loss (RL), and the absorption peaks of the composites shifted to lower frequencies as the filler content increased. The optimum amount of GS to be added was 2 wt%, and the GS-200-2% sample exhibited the lowest RL value of −31.27 dB at 7.6 GHz. The optimum concentration of the FeCo/GS composite was 3 wt%, with the lowest RL value of −20.67 dB at 7.8 GHz.

With reliable antibacterial properties, Ag can be used to prepare Ag/graphene hybrid fillers to enhance the antibacterial and other properties of rubber. Li et al. [168] prepared the polydopamine (PDA)-modified GO and then loaded Ag onto the surface of mGO to prepare Ag-PDA-GO hybrid fillers. Subsequently, the hybrid fillers were introduced into the NR latex to prepare Ag-PDA-GO/NR composite films using the dipping method. When the GO content was less than 0.1 phr, the Ag-PDA-GO sheets were uniformly dispersed in the NR matrix with a strong interfacial interaction observed between the NR macromolecules. The addition of hybrid fillers significantly improved the mechanical and gas barrier properties of the rubber composites. Particularly, the Ag-PDA-GO/NR composite exhibited excellent antibacterial properties. When the GO content was only 0.1 phr, the minimum inhibitory concentrations of *Escherichia coli* (*E. coli*) and *Staphylococcus aureus* (*S. aureus*) were 16 and 32 μg/mL, respectively.

Zinc dimethacrylate (ZDMA), another common active cross-linking agent for rubber, can improve the cross-linking density and mechanical strength of rubber. ZDMA/graphene hybrid fillers have been reported to enhance the properties of rubber. Yuan et al. [169] prepared a graphene (referred to as GN in the study [169])-ZDMA filler based on the in situ formation of ZDMA in GN layers and then mixed it with EPDM using two-roll milling. The GN sheets were homogeneously embedded in the matrix, which facilitated the uniform distribution of ZDMA. Furthermore, the GN-ZDMA constructed multiple cross-linking networks in the rubber matrix, and a synergistic reinforcing effect of GN-ZDMA was observed on the EPDM. Thus, the created GN-ZDMA significantly increased the cross-linking density and retention ratio during the aging of EPDM composites. The mechanical properties and durable sealing resilience were also enhanced. In comparison with neat EPDM, the addition of GN-ZDMA resulted in a 291% increase in tensile strength and a 48% increase in elongation at break, higher than those of EPDM/GN and EPDM/ZDMA.

Tan et al. [170] reported that barium titanate (BT)/GNP hybrid fillers can be added to fluorosilicone rubber (FSR) to prepare composites with high dielectric properties. The adequate dispersion of the GNP/BT hybrid fillers in the FSR matrix caused the FSR/GNP/BT composites to exhibit a lower dielectric loss and high dielectric permittivity. The excellent dielectric properties of these composites rendered them suitable for modern elastomer electronic devices.

As Pd can effectively absorb hydrogen gas, graphene/Pd hybrid fillers mixed with rubber can be used as a getter with excellent hydrogen absorption properties. Wang et al. [171] prepared silanized GO (SGO)/Pd/SR composites using the solution mixing method. With the addition of Pd-decorated SGO, the SR composites exhibited enhanced catalytic hydrogenation performance with excellent self-healing and processability. Therefore, these composites can be used as environmentally adaptable getters.

Magnetic Fe particles can be coated with graphene mixed with rubber to prepare magnetorheological elastomers with reliable damping properties. Additionally, Fe-coated graphene can enable the composites to efficiently handle both high- and low-frequency vibrations. Zhang et al. [172] fabricated a Fe-coated graphene filler (G-Fe) by using Fe (CH_3_COO)_2_·4H_2_O as the precursor and glucose as the reducing agent. The hybrid fillers were mechanically mixed with carbonyl iron powder (CIP) and NR to obtain the magnetorheological elastomers. The results indicated that G-Fe was reliably compatible with the rubber matrix. With the addition of G-Fe, the composites exhibited enhanced magnetorheological effects and thermal oxygen aging performance, rendering them suitable for use in the construction of tank shock absorbers.

Furthermore, boron nitride (BN) [173,174,175], platinum (Pt) [176], zwitterionic chitin nanocrystal (NC) [177], nano-diamond (ND) [178], lignin [179], chopped glass fiber, fibrous sepiolite [180], collagen(COL) or gel [181,182], molybdenum disulfide (MoS_2_) [183], electrolyte–iron particles (EIP) [184], tetrapod zinc oxide whisker (T-ZnOw) [185], SiC [186,187], and methyl methacrylate(MMA) [188,189,190] have been mixed with graphene to prepare graphene hybrid fillers for reinforcing rubber. Combining their own unique properties can enhance the thermal conductivity, fuel catalytic activity, biocompatibility, mechanical properties, thermal stability, abrasion resistance, and dielectric properties in the rubber material.

Table 6 summarizes the preparation methods, improved properties, and applications of other graphene (graphene derivatives)/hybrid filler composites, including the graphene hybrid filler type and rubber matrix type.

### 2.7. Ternary Graphene-Based Hybrid Fillers

In addition to binary graphene (graphene derivatives)-based hybrid fillers, ternary graphene (graphene derivatives)-based hybrid fillers have also been used to improve the properties of rubber composites.

Wang et al. [192] fabricated non-covalently modified rGO-CNT hybrid fillers via electrostatic assembly, which were used to grow SiO_2_ nanoparticles in situ to obtain three-phase rGO-CNTs-SiO_2_ fillers. The rGO-CNTs-SiO_2_/hydrogenated nitrile butadiene rubber (HNBR) was prepared using direct mechanical mixing, and the three components promoted dispersion and reduced the entanglement of the CNTs and the stacking of the rGO in the rubber matrix. When the mass ratio of rGO:CNTs was 1:5 and the mass percentage of rGO/CNTs was 2%, rGO-CNTs-SiO_2_ exhibited the best reinforcement performance of static and dynamic mechanical properties. The maximum tensile strength of the rGO-CNTs-SiO_2_/HNBR composites reached up to 30.1 MPa, indicating that three-phase graphene hybrid fillers have potential applications in high-performance rubber composites. Wei et al. [193] examined the addition of GO and CNT hybrid fillers to CB/NR composites for preparing GO/CNT/CB/NR composites using the latex mixing method. The addition of GO/CNT hybrid fillers improved the dispersion of CB in the rubber matrix. Thus, a more stable and efficient network in the NR improved the fatigue crack growth resistance and reduced heat build-up in the NR composites.

Sun et al. [194] conducted an interesting study using an elastic rubber band to prepare a superhydrophobic strain sensor mixed with CNTs, rGO, and hydrophobic fumed silica (Hf-SiO_2_). The addition of CNTs and rGO layers endowed the sensor with an extremely low detection limit (0.1%), high sensitivity, and a fast and reliable response to different signal changes. Hf-SiO_2_ sensors can be used to fabricate composites with reliable super-hydrophobicity, self-cleaning properties, and corrosion resistance. Therefore, composites with three-phase hybrid fillers can be used as high-performance strain sensors in next-generation intelligent wearable electronics.

CB and silica are common reinforcing fillers that are widely used in the rubber industry. A three-phase CB/SiO_2_/GE hybrid filler has also been used to improve the mechanical properties of rubber [195,196,197,198]. Yu et al. [196] analyzed the effects of different filling ratios of CB/GE/SiO_2_ on the mechanical properties of NR composites using orthogonal experiments. The results indicated that when the mass fractions of CB, GE, and SiO_2_ were 41, 4, and 1 g, respectively, the maximum true strain in the mechanical properties of NR was 139.8%. Additionally, this filler content endowed the NR composites with a maximum true stress of 85.695 MPa. To enhance the dispersion of CB and silica in the NBR matrix, Qiu et al. [198] fabricated GO/silica hybrid fillers that anchored ultrasmall mercaptopropyl-doped silica (HS@SiO_2_) on a GO nanosheet. The hybrid fillers were then mixed with latex, NBR, and CB to obtain HS@SiO_2_/GO/CB/NBR composites. The morphology of the silica changed from spherical particles to nanosheets and was better anchored on the GO sheets, which were physically cross-linked with rubber molecules through mercaptopropyl and NBR molecules. Consequently, the HS@SiO_2_-GO/CB/NBR composites exhibited a higher cross-linking density and interfacial interaction between the filler and rubber than the SiO_2_/GO/CB/NBR composites. This significantly increased the tensile strength of the CB/NBR composites from 8.9 to 16.1 MPa with only 1 phr HS@SiO_2_-GO.

Owing to its unique surface structure, ND exhibits excellent thermal conductivity and stability. Therefore, it can be used as a suitable intercalation material for GO and to facilitate rubber vulcanization. Yang et al. [199] prepared mGO/ND/nano-ZnO hybrid fillers using the latex mixing method to improve the vulcanization efficiency of NR composites. The ND was grafted onto graphite oxide with the aid of 4,40-methylene diphenyl diisocyanate (MDI), and nano-ZnO was loaded onto the mGO/ND complex using a wet chemical method to synthesize the mGO/ND/nano-ZnO functional hybrid fillers. With high surface activity and favorable dispersity in the rubber matrix, the hybrid fillers remarkably promoted the vulcanization characteristics of NR and reduced its optimum curing time. When 1 wt% nano-ZnO was loaded onto the mGO/ND complex, the activation energy of the rubber composite decreased by 16% during the cross-linking period, and the vulcanization efficiency was improved by approximately 63%.

TiO_2_ nanoparticles exhibit excellent stability, thermal properties, and chemical resistance, whereas nickel (Ni) nanoparticles are known to impart adequate dielectric and magnetic properties to NR. Therefore, GE/TiO_2_/Ni hybrid nanoparticles have been used to reinforce NR. Mathew et al. [200] used direct mechanical mixing to prepare GE/TiO_2_/Ni/NR composites. Uniformly distributed nanofillers in the NR matrix significantly improved the mechanical properties of rubber composites, such as strength, Young’s modulus, and elasticity. An enhancement in the mechanical properties (yield and tensile strengths increased by 94 and 147%, respectively) was achieved using rGO and TiO_2_ nanoparticles with a composition of 1.25 wt%. A similar improvement was observed when TiO_2_ and Ni nanoparticles with a composition of 0.1 wt% were introduced into the elastomer by maintaining the same composition of rGO. Furthermore, the blending of nano-sized SiO_2_ additives and TiO_2_ in EPDM increased the thermal stability and electrical performance of EPDM composites [201]. Azizi et al. [202] prepared EPDM and silicone hybrid rubber composites by mechanically mixing modified fumed silica (MFS), TiO_2_, and graphene. The addition of the MFS/TiO_2_/GE hybrid fillers increased the dielectric constant, alternating current dielectric breakdown strength, thermal stability, and thermal conductivity of the composites. Therefore, EPDM rubber composites with MFS, TiO_2_ additives, and low graphene content yielded appropriate thermal and electrical performances of the compounded composites used in outdoor insulating applications.

Aluminum trihydroxide (ATH) is used as a flame retardant in rubber. ATH, graphene, and CB hybrid fillers have been reported to enhance rubber properties as well. Zirnstein et al. [203] studied multilayer graphene (MLG) with only 10 graphene sheets to replace parts of CB and ATH in HNBR composites for improving the mechanical properties and fire retardancy of HNBR and reducing the filler content. Six HNBR rubber mixtures with systematic variations in the CB, MLG, and ATH fillers were investigated, and the composites were prepared using a two-step process. Initially, the masterbatch was prepared by dispersing MLG in a toluene/HNBR solution, which was then mixed with other ingredients using two-roll mixing. When 3 phr MLG replaced 15 phr CB, 3 phr ATH, or 15 phr CB + 3 phr ATH, a considerable and consistent improvement was observed with respect to the curing, rheological, and mechanical properties of HNBR. Additionally, MLG achieved advanced flame retardancy by improving the protective layer, which acted as an afterglow suppressor during the burning phase. HNBR/CB20/ATH47/MLG, which resulted from the substitution of ATH with MLG, exhibited the best mechanical and flame-retardant performance in the composites. ATH, milled glass fibers (MGFs), and graphene hybrid fillers are also known to improve the fire retardancy of composites. Nazir et al. [204] fabricated ATH/MGFs/GNPs/SR composites using the solution mixing method. The hybrid fillers improved fire retardancy and limited the oxygen index of the SR composites. Particularly, GNPs and MGFs collectively improved the tensile strength compared to the SR composites that contained only ATH.

The synergistic effect of MWCNTs, graphene, and Fe_3_O_4_ magnetic particles in the rubber matrix can improve the response of the changes in the electrically conductive channel to external strain. This resulted in composites with excellent sensing properties. Guo et al. [205] prepared MWCNTs/GE/Fe_3_O_4_/SR composites with biomimetic scorpion foot-slit microstructures. The special structure endowed the composites with outstanding stretchability, high sensitivity (GF = 100, 150% to 160% strain), rapid response/recovery times (approximately 100 and H160 ms), extremely low strain detection limit (0.16% strain), and high durability after 9000 cycles at 10% strain. Consequently, the composites could accurately recognize changes in external signals generated by different human beings. These composites can be applied in wearable electronics and other intelligent electronic devices.

The preparation of vertically aligned graphene hybrid fillers in a rubber matrix can significantly increase the electricity consumption of the composites. Song et al. [206] fabricated vertically aligned CNTs/GE/CNF hybrid filler network foams using the ice-template assembly method, which were then filled with SR to obtain the composites. CNF in aqueous dispersions served as an adhesive to link the CNT with GE, enhancing the hybrid fillers further. The addition of three-phase vertically aligned hybrid fillers significantly increased the compressive strength and electrical conductivity of the composites. This endowed the composites with a low conductive percolation threshold and excellent piezoresistivity.

Carboxymethyl chitosan (CMCS) is another polymer known for its antibacterial properties. Su et al. [207] manufactured antibacterial NR latex gloves using GO/CMCS/Ag hybrid fillers. CMCS was used as a cross-linking agent, which increased the interfacial interactions between GO and the rubber matrix. Additionally, it could construct a special 3D network in rubber. With the addition of these hybrid fillers, NR composites exhibited improved mechanical, barrier, and antibacterial properties against *E. coli* and *S. aureus*. Owing to their excellent properties, these composites can be used in medical gloves.

Overall, the ternary graphene hybrid fillers in rubber composite materials combine the advantages of various fillers, allowing rubber composites to diversify their applications. Moreover, using multiple fillers effectively achieves a good balance between performance and the cost of rubber composites. However, in a ternary filler system, the challenge lies in better controlling the synergistic effect and dispersion of these various fillers in the rubber matrix. This is more challenging than with binary fillers and requires careful consideration. Table 7 summarizes the preparation methods, improved properties, and applications of different three-phase graphene hybrid filler rubber composites, including the graphene hybrid filler type and rubber matrix type.

## 3. Approaches for Performance Enhancement of Rubber with Graphene-Based Fillers

The effectiveness of the reinforcement provided by graphene (graphene derivatives)-based hybrid fillers to the rubber matrix is a critical aspect for improving the properties of rubber composites. The dispersion of graphene and other fillers, the strong interaction between graphene hybrid fillers and the rubber matrix, and the synergistic effect of graphene (graphene derivatives) and other fillers are the three prominent factors that determine the reinforcement of rubber composites. To consider the aforementioned factors, several improvement measures have been incorporated in the preparation of graphene (graphene derivatives)-based hybrid fillers in rubber composites, which are summarized in this section.

### 3.1. Enhanced Preparation Methods

In the case of graphene (graphene derivatives)-based hybrid fillers in rubber composites, the preparation method determines the dispersion of hybrid fillers and the filler–rubber interaction. Therefore, the selection of an appropriate preparation method is crucial. The masterbatch method is commonly used to prepare rubber composites with graphene/CB or graphene/silica hybrid fillers [34,35,42,46,62,63]. This method involves first preparing a latex- or solution-based GE/rubber masterbatch and then mechanically mixing it with other fillers. This can improve the dispersion of graphene in the rubber matrix; however, the high viscosity of the graphene/rubber masterbatch may generate dispersion problems and hinder the processing of CB or silica in the rubber matrix, particularly at high concentrations. Therefore, a new preparation method using high CB or silica content was considered necessary for significantly improving the performance of graphene hybrid fillers in rubber composites. Wang et al. [41] developed a modified latex process, wherein the wet compounding process was combined with ultrasonically assisted latex mixing (WCL) to prepare rGO/CB/NR composites. Mixing CB and graphene in an aqueous phase improved the dispersion of CB in the presence of highly concentrated graphene-based rubber composites. In comparison with the rGO/CB/NR composites obtained using the common masterbatch method, the composites prepared via the WCL method exhibited better mechanical properties and a lower value of tan(δ) at 60 °C with the same rGO and CB content. With an rGO content of 0.5 phr, the tensile strength of the composite prepared using the WCL method was approximately 28.3 MPa, and the elongation at break was nearly 733%; these values were higher than the tensile strength (approximately 25.3 MPa) and elongation at break (approximately 632%) of the composites prepared via the masterbatch mixing method. Furthermore, the addition of rGO to the CB/NR system improved the hardness (adding 8 phr rGO increased the hardness by nearly 71%), thermal conductivity (adding 8 phr rGO increased the thermal conductivity by 33%), and thermal oxygen aging resistance of the composite. These improvements can significantly affect the practical application of rubber composites.

### 3.2. Controlling of 3D Networks of Graphene Hybrid Fillers

The construction of special 3D network structures of graphene hybrid fillers is another important strategy for enhancing the performance of graphene (graphene derivatives)-based hybrid fillers/rubber composites. In addition to the composites prepared by latex mixing, constructing an effective 3D porous conductive graphene hybrid filler skeleton, such as an aerogel or foam, followed by injecting the rubber matrix into the 3D scaffold, can be an effective method for preparing composites with special structures. Moreover, this method can construct a highly effective 3D network in the rubber matrix owing to its specific porous structure and lightweight samples, which significantly improves the electrical conductivity, thermal conductivity, EMI-shielding, and microwave-absorption properties of rubber composites [123,150,159].

**Figure 4:**
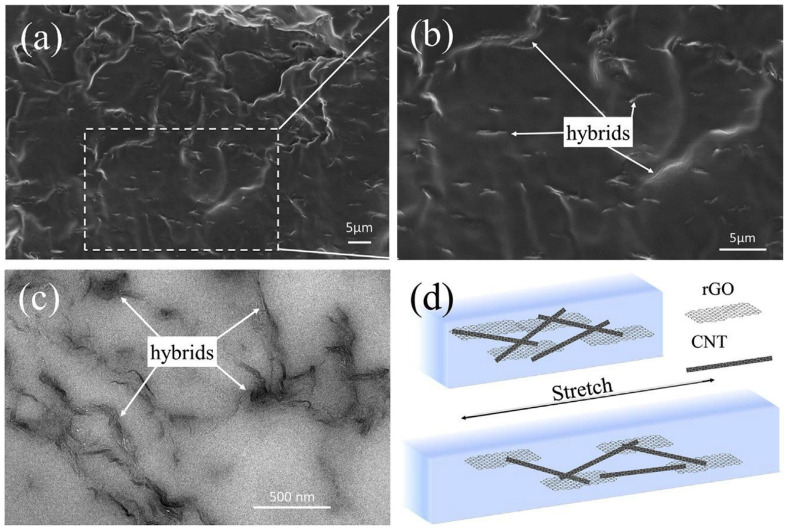
(**a**,**b**) SEM and (**c**) TEM images of SBR/rGO-CNT composite and (**d**) schematic diagram of the redistribution of the hybrids in the SBR composite under tensile process (reprinted/adapted with permission from Ref. [102], 2024, Elsevier).

Several researchers have reported 3D graphene/CNT/rubber composites [28,102,118,119,120,121,210]. Liu et al. [28] prepared anisotropic graphene/MWCNT hybrid fillers based on the KOH-induced hydrothermal reaction and heat treatment graphitization at 2800 °C to obtain hybrid aerogels with 3D continuous networks. Subsequently, they blended it with SR to fabricate a lightweight dual-function integrated composite. The specific 3D network structure endowed the composite with excellent thermal conductivity and electromagnetic shielding properties. When the ratio of GO to MWCNTs was 1:3, the thermal conductivity of the composite reached 1.30 W·m^−1^·K^−1^ with a low filler content (2.77 wt%), which was 465% higher than that of the pure SR (0.23 W·m^−1^·K^−1^). The composite exhibited the maximum electric conductivity with an EMI SE of 42 dB in the K-band. Liu et al. [102] fabricated rGO-CNT in SBR composites by emulsion blending, followed by the freeze-drying process. Here, 1D CNTs acting as spacers were inserted between the 2D rGO sheets, which effectively prevented the restacking of graphene sheets and the agglomeration of CNTs. The hybrid fillers composed of CNT and rGO exhibited excellent dispersion in the SBR composite, thereby facilitating the construction of 1D and 2D interconnected networks in the SBR composites (Figure 4). This network could be deformed under low-strain-constructed rGO sheets based on the connections of CNTs, which significantly enhanced the electrical conductivity of the composites even under low tensile strains. Jia et al. [120] prepared flexible and electromagnetic-shielding graphene/MWCNTs/PDMS composites. The composites were prepared using a simple direct freeze-casting method, followed by carbonization from 1200 to 1600 °C and graphitization at ultra-high temperature (2800 °C); this resulted in oriented pore 3D network structures in the composites. Such specific structures can significantly enhance the thermal conductivity, mechanical properties, and electromagnetic shielding performance of composites. Particularly, the corresponding total SE of GC1400 (GO/CNT foams annealed at 1400 °C)/PDMS was as high as 54.43 dB. The specific SE was 87.86 dB·cm^3^/g in the X-band when 0.98 wt% fillers were loaded.

**Figure 5:**
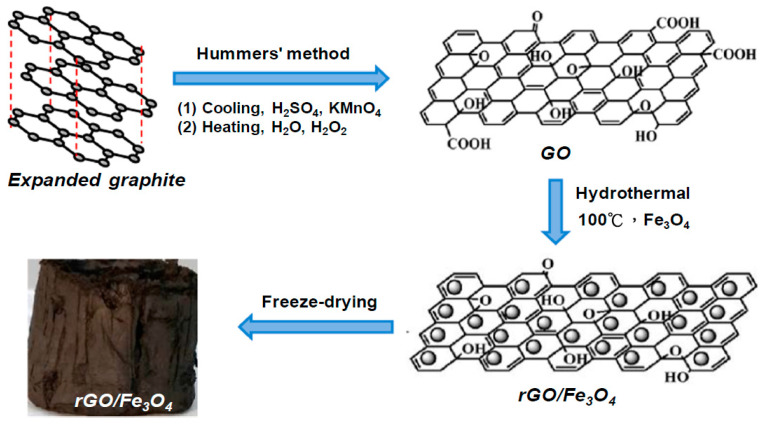
Experimental procedure for the synthesis of rGO/Fe_3_O_4_ composites (reprinted/adapted with permission from Ref. [146], 2024, Elsevier).

EMI-shielding and microwave-absorption properties are the most important aspects of rubber-based electronic devices. The preparation of special 3D network graphene/rubber composites with the addition of magnetic particles, such as Fe_3_O_4_, can significantly improve these properties of rubber composites, increasing their application in electronic products. Zhu et al. [211] assembled an innovative Fe_3_O_4_/graphene foam/PDMS material by anchoring magnetic Fe_3_O_4_ particles onto a highly electronically conductive 3D graphene foam. Owing to the synergistic effect between Fe_3_O_4_ nanoparticles and the graphene foam, the EMI SE of the composite (approximately 1.0 mm) increased from approximately 26.6 dB to 32.4 dB in the frequency range of 8.2–12.4 GHz for the graphene foam/PDMS composite. Furthermore, after 10,000 repeated cyclic bends, the EMI SE of the Fe_3_O_4_/graphene foam/PDMS composite was 29.4 dB. Wu et al. [146] also synthesized 3D porous-network composites containing rGO and Fe_3_O_4_ nanoparticles (rGO/Fe_3_O_4_) using hydrothermal and freeze-drying processes (Figure 5). The transmission electron microscopy (TEM) results indicated that Fe_3_O_4_ nanoparticles were effectively deposited on the 3D porous network structure of rGO. The Fourier transform infrared and X-ray photoelectron spectroscopy results verified that the Fe_3_O_4_ nanoparticles were intercalated within the rGO and connected to the rGO through C–O–Fe bonds. Therefore, the hybrid 3D rGO/Fe_3_O_4_ hydrogel fillers mixed with thermo-plastic SR (RTV-615) significantly improved the microwave-absorption properties. For the rGO/Fe_3_O_4_/RTV-615 composite with the weight ratio of 2:1 for rGO: Fe_3_O_4_, the minimum reflection loss reached −31.3 dB, and the effective absorption bandwidth reached 6 GHz in the range of 2–18 GHz when the absorber was 2.0 mm thick.

The construction of a special 3D network with the addition of graphene hybrid fillers can significantly increase the thermal conductivity of rubber composites. An et al. [174] used modified BN to react with rGO. Here, the carboxyl activator (EDC) and catalyst (NHS) were used to connect BN and rGO via an amidation reaction. The obtained hybrid fillers were then treated with liquid nitrogen to obtain 3D networks for the BN/rGO foam. BN/rGO/NR composites with a 3D network were obtained by introducing NR into the BN/rGO foam. With a special 3D network and a stronger combination of rGO and BN, the interfacial thermal resistance and phonon scattering could be reduced. Therefore, a higher through-plane thermal conductivity of 1.28 W·m^−1^·K^−1^ was achieved, as well as satisfactory electrical insulation at a low filler loading of 4.9 vol%. Song et al. [159] fabricated cellulose carbon aerogel@rGO (CCA@rGO) using vacuum impregnation, freeze-drying, and thermal annealing. The hybrid aerogels were backfilled with PDMS to obtain composites with a 3D network. Owing to the special 3D network, the composites exhibited a high EMI SE of up to 51 dB, which was 3.9 times higher than that of the CCA/rGO/PDMS EMI composites without a 3D network (13 dB) with the same filler loading. Furthermore, the special network endowed the composites with high thermal stability and an excellent thermal conductivity coefficient (λ = 0.65 W·m^−1^·K^−1^).

### 3.3. Surface Functionalization and Interface Improvement

In graphene-based hybrid filler systems, obtaining a higher interaction between the graphene hybrid fillers and rubber matrix is essential for obtaining high-performance rubber composites, particularly in the case of high-filler-content systems.

**Figure 6:**
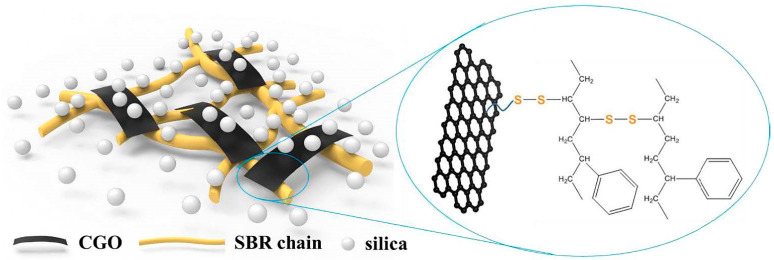
The proposed interfacial reaction between CGO and SBR matrix through disulfide exchange reactions in CGO/SiO_2_/SBR composites (reprinted/adapted with permission from Ref. [91], 2024, Elsevier).

The modification of graphene and other fillers is an effective method of improving these interactions. In general, mGO can react chemically or generate strong electrostatic interactions with the rubber matrix, thereby enhancing the interaction between the rubber and graphene hybrid fillers. Wang et al. [91] realized the simultaneous reduction and chemical graft functionalization of GO (CGO) by using CDHC, a novel functional agent with a dynamic disulfide bond. This was followed by the preparation of SiO_2_/CGO/SBR composites with a high silica content (60 phr) using the latex mixing method. The performance of the SiO_2_/CGO/SBR composites was compared with that of the hydrazine hydrate reduced GO (HGO)/SiO_2_/SBR composites; here, the HGO was prepared using the conventional reducing agent, hydrazine hydrate. The results indicated that CDHC grafted onto the GO surface participated in the vulcanization of SBR through a dynamic sulfur–sulfur bond exchange reaction (Figure 6). This generated a close interfacial interaction between the CGO and rubber matrix. CDHC-reduced GO can be uniformly and stably dispersed in the aqueous phase as well, providing a prerequisite for preparing graphene-based rubber composites by latex mixing. Owing to excellent dispersion and close interface interaction, simultaneous reinforcement and toughening of SBR composites with a high silica content (60 phr) were achieved by introducing the novel CGO. In addition, compared to the GO reduced by hydrazine hydrate, the nanocomposites containing CGO exhibited better mechanical properties and thermal conductivity. This verified that the simultaneous reduction and chemical graft functionalization of GO can effectively improve the performance of graphene-reinforced rubber composites. Furthermore, 3(Mercaptopropyl)trimethoxysilane (MPTMS) is an effective modifier for producing sulfhydryl groups on GO. Zhang et al. [73] reported that MPTMS-modified GO can be used to initiate the thiol-vinyl click reaction with the rubber matrix during the vulcanization process. Subsequently, the mGO network cross-linking points were constructed in solution-polymerized SBR (SSBR). This resulted in stronger chemical interfacial interactions between the filler and composites, further improving the rolling resistance and energy-saving properties of the composites with the addition of 65 phr silica. This excellent property renders the composite more suitable for use in energy-saving green tires. The addition of a third component that exhibits a strong interaction with both the graphene hybrid fillers and rubber matrix can significantly enhance the interfacial interaction between the filler and rubber matrix. Zhang et al. [208] used γ-aminopropyltriethoxysilane to simultaneously modify GO and silica to obtain positively charged fillers. The negatively charged maleic anhydride (MAH) hydrolysis was electrostatically assembled with positive GO and SiO_2_ to obtain the composite particles (NG-NS) with “bridged structures.” The vinyl groups of MAH in NG-NS reacted with the vinyl groups of SBR during vulcanization, thereby improving the interfacial interaction between the hybrid fillers and the SBR matrix. Thus, the NG-NS/SBR composites exhibited an improved modulus (300%), tensile strength, and abrasion resistance. Moreover, the composites had a lower heat build-up than the GO/SiO_2_/SBR composites.

**Figure 7:**
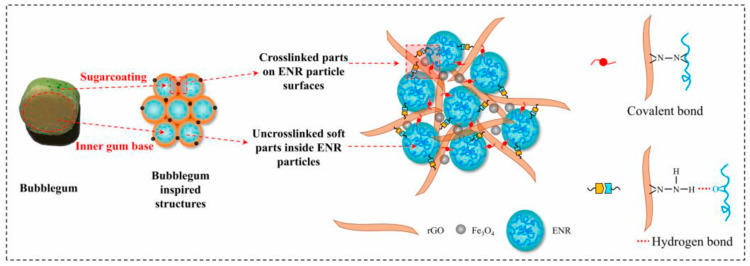
Illustration of the interfacial interaction between rGO and ENR in oENR: covalent and hydrogen bonds (reprinted/adapted with permission from Ref. [147], 2024, Elsevier).

Owing to the special structure of bubblegum, the higher interaction between the hard sugar coating and the inner gum can endow the bubblegum with suitable properties. Graphene-based hybrid fillers in rubber composites can be used to construct a bubblegum structure to obtain a higher filler–matrix interaction. Zhu et al. [147] constructed N–C covalent and NH–O hydrogen bond interfacial interactions between rGO and an epoxidized natural rubber (ENR) matrix using hydrazine, which acted as a bridge in the ENR/rGO/Fe_3_O_4_ composites (Figure 7). The double bond interactions resulted in a “hydrazine bridge”, which was identical to the hard-sugarcoating function observed in bubblegum. This in turn efficiently enhanced the ENR composites. The strong interfacial interaction between the hybrid fillers and rubber endowed the composites with excellent mechanical, electrical, magnetic, and sensing properties, thereby rendering them useful as multifunctional materials.

## 4. Reinforcing Mechanism of Hybrid Graphene Fillers for Rubber Composites

Understanding the reinforcing mechanism of graphene (graphene derivatives) in rubber composites is crucial as it can aid in improving the fabrication processes.

**Figure 8:**
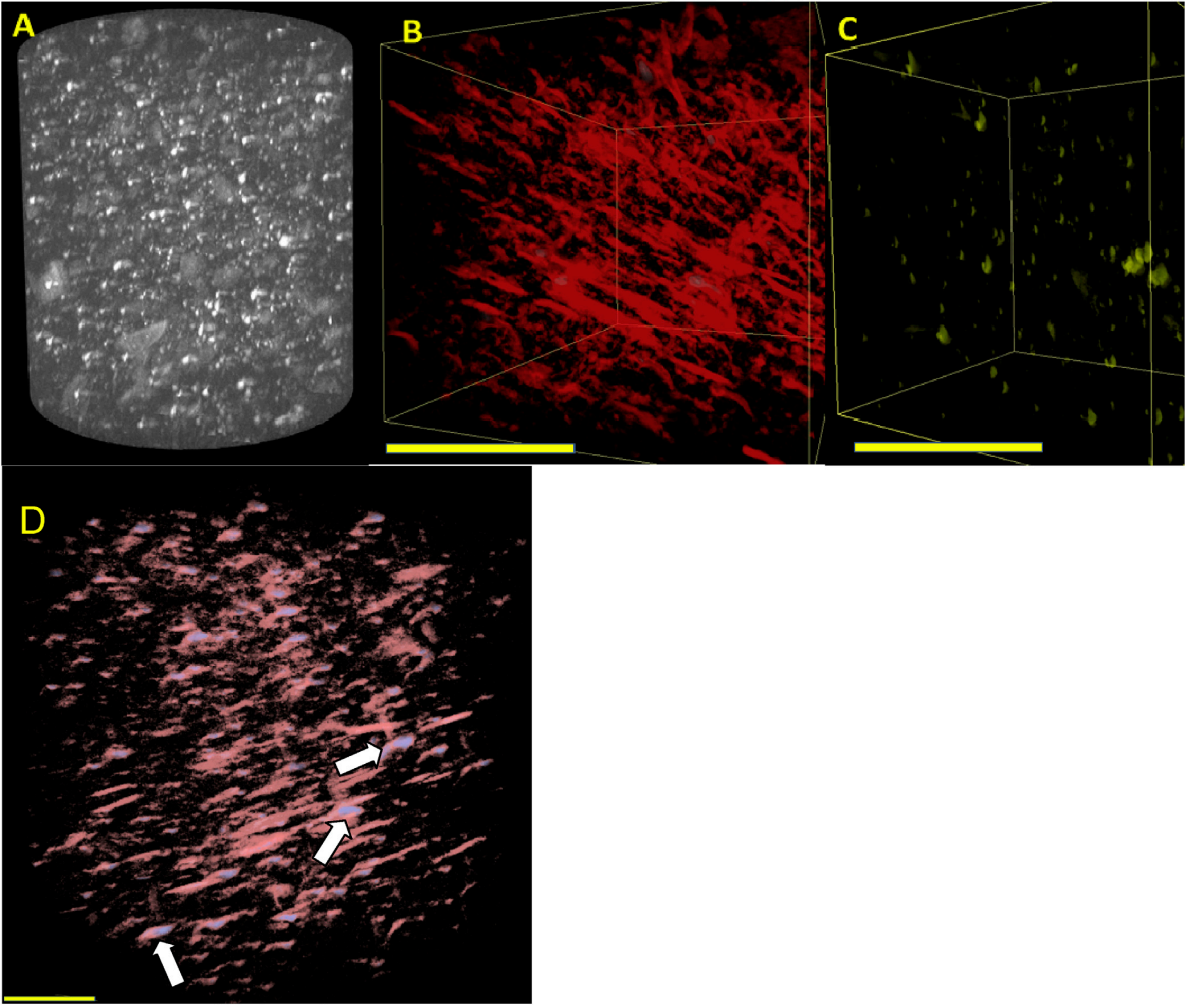
(**A**) Total cylindrical volume of the EPDM-6 sample (348 mm in diameter and 360 mm in height); (**B**,**C**) detail of segmented m-CT images showing the distribution of the GNPs and CB aggregates, respectively (scale bar is 50 mm). (**D**) Detail of segmented m-CT images showing the whole distribution of the GNPs (in red) and CB aggregates (in blue indicated by the arrows) (scale bar is 50 mm) (reprinted/adapted with permission from Ref. [36], 2024, Elsevier).

Several studies have focused on the mechanism through which graphene/CB hybrid fillers reinforce rubber. For an in-depth understanding of material behavior, Das et al. [212] utilized 3D-TEM to qualitatively characterize the complex filler morphology of a CB/graphene/SSBR rubber matrix. The study explored the use of 3D tomography and 3D-TEM to investigate the dispersion of stacked graphene sheets in rubber composites. The results showed that 3D tomography was an effective method for detecting and distinguishing individual objects, including different filler platelets. The presence of CB was found to facilitate the dispersion/exfoliation of stacked graphene sheets into individual sheets. The study also detected the presence of oligo-layer graphene sheets using 3D-TEM, particularly in the complex morphologies caused by filler–filler networks in all spatial dimensions of the rubber matrix. These findings contribute to the understanding of the behavior of graphene in rubber composites and have potential applications in the development of high-performance rubber materials. Valentini et al. [36] analyzed the synergistic reinforcement effect of graphene nanosheets (referred to as GNPs in the study [36]) and CB in an EPDM matrix. The nanocomposites were prepared using the mechanical mixing method, with a sufficient number of GNPs and CB dispersed in the EPDM matrix. This increased the thermal conductivity, damping strength (shock absorption properties), and mechanical properties of the nanocomposites. As the replacement of CB with GNPs reduced the CB aggregation, the percolation of the hybrid fillers and the interface resistance of the composites were improved. Figure 8 depicts the mean computed tomography image of EPDM with 1 wt% GNP and 24 wt% CB. As indicated in the figure, GNP and CB were uniformly dispersed in the polymer matrix. The formation of CB aggregated on the GNP surface (Figure 8) realized additional conductive paths and interfacial resistance in the CB/GNP/EPDM composites.

**Figure 9:**
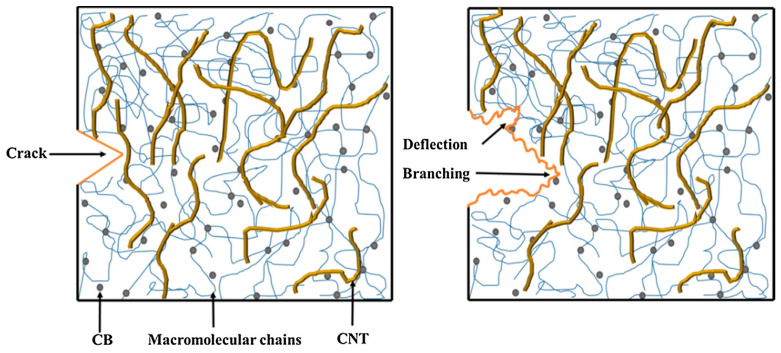
Diagrammatic representation of relation between fatigue and microstructures of the composites (reprinted/adapted with permission from Ref. [42], 2024, Elsevier).

**Figure 10:**
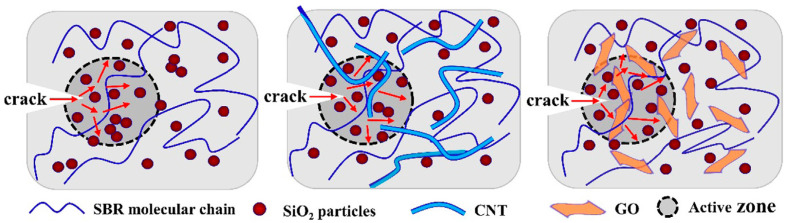
Schematic of crack propagation for SBR composites (reprinted/adapted with permission from Ref. [92], 2024, Elsevier).

Fatigue properties are also important for rubber composites. Guo et al. [42] prepared GO-CB/NR composites by mechanically mixing a GO/NR masterbatch with CB and focused on their strain-induced crystallization, fatigue, and durability properties. The results indicated that the addition of GO inhibited the agglomeration of CB, which was beneficial for increasing the internal friction and hysteresis loss. Particularly, the large structure of the GO sheets led to a stronger interaction with rubber molecules than with spherical CB particles, resulting in high internal friction and hysteresis. This in turn dissipated the tearing energy and reduced the number of cracks during the cyclic loading process. Additionally, the crack tips of the GO-CB/NR exhibited branched and deflected morphologies, similar to a checkerboard, during the crack propagation phase. Consequently, higher fatigue lives were observed under uniaxial and multi-axial cyclic loading than those observed in the CB/NR composites (Figure 9). Xu et al. [92] examined the effect of GO and SiO_2_ hybrid fillers on the fatigue properties of SBR composites under both uniaxial and multi-axial conditions. The results indicated that the SBR composites with only SiO_2_ particles exhibited a weak interface between the filler and SBR matrix, which caused cracks to effortlessly bypass the SiO_2_ particles. This reduced crack propagation and worsened the fatigue properties. The introduction of GO to the SiO_2_/SBR composites resulted in a high-filler-content network. The lamellar GO sheets were oriented perpendicular to the direction of crack propagation, which inhibited crack propagation and reduced the rate of crack growth. This significantly improved the fatigue properties of SBR composites. Figure 10 illustrates the schematics of crack propagation for the different SBR composites.

**Figure 11:**
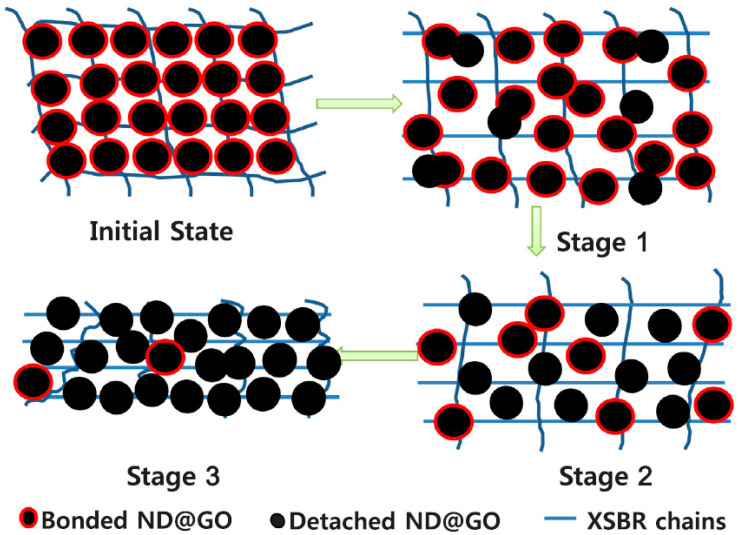
Schematic representation of filler–rubber network variation during external force application (reprinted/adapted with permission from Ref. [178], 2024, Elsevier).

The Payne effect can occur in filled rubber composites when the composites are treated with cyclic loading conditions, especially at small strain amplitudes, which can illustrate the viscoelasticity of the rubber [55,172]. The Payne effect is related to the breakdown of the filler network, the entanglement and disentanglement of polymer chains, and the yielding of a glassy layer surrounding the nanoparticles [213,214]. Thus, it can provide insight into the filler dispersion and interfacial interaction between the filler and matrix, which is essential for rubber composites with different graphene hybrid fillers [43,178,215]. Xue et al. [43] prepared different GO/CB/NR, rGO/NR/CB, and surface-modified GO (mGO)/NR/CB composites and focused on their viscoelastic and fatigue properties. The results showed that, when graphene was added to the composites, the ΔE’/E’_0.1%_, which represents the normalized Payne effect, decreased significantly. This is because, compared with CB, different graphene derivatives can provide more interface groups and interface structures to form chemical cross-link and mosaic structures with the rubber matrix, which can inhibit internal damage to the composites under amplitude loading and reduce the Payne effect. As a result, composites with graphene can have better elasticity and viscosity properties. Zhang et al. [178] investigated the Payne effect and strain sweep of an elastomeric network in ND@GO/rubber carboxylated SBR (XSBR) nanocomposites. The results verified that neat XSBR exhibited a lower initial storage modulus and a higher inflection point, where the storage modulus was lower than that of the XSBR/ND@GO compounds. This indicated that the XSBR with the hybrid fillers comprised a sophisticated and stronger network. According to a previous study, filler networks can be divided into three types with different filler contents [158]. In the case of different ND@GO filler contents, the rubber network was divided into flexible and rigid filler networks. In a flexible network, the rubber chain undergoes distinct deformation, whereas the filler network gradually breaks down until a high strain (approximately 60%) is reached. In a rigid network, the GO hybrid fillers can contact or overlap each other and reach a higher concentration, thus leading to a high initial storage modulus. When an external force is applied, the filler network breaks down, owing to the non-flexible network even at a small strain, and the modulus rapidly decreases in the initial stage. Figure 11 illustrates the results corresponding to this observation.

The entanglement-bound rubber tube (EBT) model can achieve the proper separation of chemical cross-links and physical constraint contributions, thereby reliably determining the cross-link densities in rubber [216,217]. This can aid in realizing the reinforcing effect of graphene in rubber materials, particularly with other fillers, at the molecular level [41,51]. Wang et al. [41] used the EBT model to demonstrate the reinforcement mechanism of graphene in rGO/CB/NR composites. The results indicated that with the addition of a high-aspect-ratio graphene sheet to CB/NR, the hybrid fillers could generate a stronger interaction with the rubber chains. Therefore, rubber limited the formation of topologically constrained structures. In the transition zone, the kinetic energy of the molecular chain increased as the distance from the filler increased. When the distance was sufficiently large, the molecular chains became free and unrestricted. Therefore, a stronger interaction decreased the lateral tube dimensions, area of the transition zone, and mean number of statistical segments between two successive entanglements. Consequently, the graphene sheet could contribute more cross-links and topological constraints, which significantly improved the mechanical properties of the CB/NR composites.

## 5. Conclusions and Perspectives

In this study, we examined the development of graphene-based hybrid fillers/rubber composites made using different preparation methods. The other fillers can compensate for the potentially inadequate performance of graphene, thereby endowing rubber materials with improved properties and wide applications. Furthermore, enhanced preparation methods, controlling the 3D networks of graphene hybrid fillers in the rubber matrix, and interface improvement between the graphene and rubber matrix can effectively facilitate the enhancement effect of graphene in the hybrid fillers. The addition of graphene to the rubber matrix with other fillers can contribute more cross-links and topological constraints, generating stronger interfacial interactions between the filler and rubber matrix in comparison with fillers without graphene (graphene derivatives). This can significantly enhance the mechanical properties, electrical conductivity, thermal conductivity, EMI shielding, and fatigue resistance of the rubber composites.

In recent years, many graphene-based hybrid fillers/rubber composites have been developed and have attracted great attention from the academic and industrial community. Based on existing research trends, the future development of graphene-based hybrid fillers rubber/composites can focus on the following aspects:

1. Diversification of applications: Now it is the opportune moment to seize the significant decline in the cost of preparing graphene and its derivatives. This presents an opportunity to create graphene hybrid filler rubber composites with excellent properties and break away from the constraints of traditional rubber materials. The application of composites can be extended from traditional fields (such as tires and shoes) to more modern areas (such as healthcare and artificial intelligence) and extended from singularity to multi-dimensional development.
2. Optimization of the fillers’ properties and the composites’ structures: There is also some room for improvement in increasing the fillers’ performance and the structure design of the rubber composites. In this way, rubber composites with more reliable and excellent properties can be created. For instance, combining graphene magnetic hybrid fillers with specific structures, such as gradient structures, layer-by-layer structures, or porous structures, can be an effective method for enhancing material absorption while reducing reflection. This is particularly significant for the preparation of “green” EMI-shielding materials with low reflection and high absorption [218,219,220].
3. Diversified rubber matrices: Exploring the blending of the rubber matrix with other materials, such as chitosan, gelatin, polyurethane, and epoxy, can endow the composites with more advantages. By combining a broader range of materials, graphene hybrid fillers/rubber composites can be used in high-end precision applications with excellent properties.

## Figures and Tables

**Table 1:** Summary of graphene (graphene derivatives)/carbon black rubber composites.

Hybrid Fillers	Rubber Matrix	Preparation Methods	Improved Properties	Application	Year and Reference
GNPs/CB	SR	Solution mixing	Electrical conductivity and piezoresistivity	Piezoresistive sensors	2014 [33]
GO/CB, rGO/CB	NR	Preparing the GO/NR and rGO/NR composites based on latex mixing, followed by mechanical mixing with CB and rubber	Static and dynamic mechanical properties, flex cracking resistance		2015 [35]
rGO/CB	SBR	Solution mixing to initially prepare the rGO/CB, followed by mechanical mixing with SBR	Electrical conductivity and tensile properties		2015 [34]
l-GFs/CB, GO/CB	SBR	Preparing the masterbatch using latex mixing, followed by mechanical mixing with CB and rubber	Mechanical, thermal, electrical, and gas barrier properties		2015 [46]
GNPs/CB	SR	Solution mixing	Electrical and mechanical performance	Flexible pressure sensor	2015 [47]
MLG/CB	CIIR	Preparing the MLG/rubber masterbatch using latex mixing, followed by mechanical mixing with CB and rubber	Mechanical properties and weathering resistance		2016 [45]
GNPs/CB	EPDM	Mechanical mixing	Thermal conductivity, damping properties, and mechanical properties		2016 [36]
GF/CB	PDMS	Solution mixing	Thermal conductivity and storage modulus		2016 [49]
GNPs/CB	SR	Solution mixing	Sensitivity, stability, and compressibility	Piezoresistive sensors	2017 [56]
GO/CB	NR	Preparing the GO/NR masterbatch initially by latex mixing firstly and then performing mechanical mixing with CB and rubber	Dynamic and static mechanical properties, heat buildup		2017 [48]
GNPs/CB	NBR	Solution mixing	Mechanical properties, electrical properties, and thermal stability		2017 [37]
GE/CB GE/SiO_2_	ENR	Mechanical mixing	Dynamic properties and wet grip		2017 [58]
GE/CB	NR	Mechanical mixing of the GE/NR masterbatch with CB and NR	Reduce heat build-up, accelerate aging and lifetime		2018 [39]
GNPs/CB	SR	Solution mixing	Electrical conductivity and sensing property	Strain sensor	2018 [40]
GNPs/CB	SR	Solution mixing with GNPs/CB, prefoamed, and solution mixing again with SR	Decrease percolation threshold and improve electrical conductivity	Robotic sensory systems and real-time wearable health monitoring devices	2018 [50]
rGO/CB	NR	Wet compounding process, combined with ultrasonically assisted latex mixing	Hardness, thermal conductivity, and anti-aging properties		2018 [41]
GO/CB	NR	Preparing the GO/NR masterbatch by latex mixing, followed by mechanical mixing with CB and rubber	Strain-induced crystallization, modulus at 100% strain, tear strength, fatigue, and durability		2020 [42]
GO/CB, rGO/CB, and mGO/CB	NR	Solution mixing	Fatigue resistance and dynamic mechanical property		2020 [43]
GE/CCB	SR	Solution mixing followed by mechanical mixing	Deformation recovery properties and electrical conductivity	Strain sensor	2021 [57]
GE/CB	SBR	Latex mixing	Mechanical property, specific capacitance, and magnetic hysteresis at room temperature with fairly low remnant magnetization		2021 [44]
GE/CB	SBR	Mechanical mixing	Tensile strength, modulus, and abrasion resistance		2023 [51]
GO/CBp	NR	Mechanical mixing	Decrease the metal wear		2023 [59]

GNPs: graphene nanoplatelets; GO: graphene oxide; rGO: reduced graphene oxide; l-GFs: low defect graphene flakes; GE: graphene; mGO: modified graphene oxide; CCB: conductive carbon black; CBp: thermal cracked carbon black; ENR: epoxidized natural rubber; CIIR: chlorine isobutyl isoprene rubber; GF: graphene foam.

**Table 2:** Summary of graphene (graphene derivatives)/silica rubber composites.

Hybrid Fillers	Rubber Matrix	Preparation Methods	Rubber Performance	Application	Year and Reference
rGO/SiO_2_	SBR	Electrostatic self-assembly	Mechanical properties, high wet-grip property, and low rolling resistance	Green tire materials	2016 [3]
mGO/SiO_2_	SSBR	Electrostatic self-assembly	Higher retention ratio of elongation and strength, resist ozone attacking.		2016 [78]
GO/SiO_2_	SR	Sol–gel method to prepare GO/SiO_2_ hybrid fillers firstly, followed by mechanical mixing with SR	Mechanical property		2016 [81]
rGO/SiO_2_	SR	Using the sol–gel method to prepare rGO/SiO_2_ hybrid fillers, followed by mechanical mixing with SR	Mechanical and thermal performances		2017 [82]
rGO/mesoporous silica	SBR	Preparing the rGO/mesoporous silica hybrid fillers using the electrostatic self-assembly method, followed by mixing with SBR	Thermal conductivity		2017 [72]
GO/SiO_2_	SBR	Preparing the GO/NR masterbatch by latex mixing, followed by mechanical mixing with silica and other agents	Mechanical properties and fatigue lifetimes		2017 [62]
GO/SiO_2_	SBR	Preparing the SiO_2_/GO hybrid fillers using the electrostatic self-assembly method, followed by solution mixing with SBR	Mechanical, dielectric, and thermal properties		2017 [77]
GO/SiO_2_	NBR	Using solution mixing to prepare the GO/SiO_2_ hybrid fillers, followed by solution mixing with NBR	Mechanical properties and elasticity		2018 [68]
mGO/SiO_2_	NR	Preparing the mGO/SiO_2_ hybrid fillers using the hydrogen-bonding self-assembly method, followed by mechanical mixing with NR	Thermal property, tensile Strength, and tear strength		2018 [79]
GO/mSiO_2_	NR	Latex mixing	Tensile and tear strengths		2018 [71]
GE/SiO_2_	NR	The SiO_2_ particles were uniformly decorated on the surface of graphene using the the Michael addition reaction firstly, and then mechanical mixing with NR	Tensile strength, tear strength compression set, thermal conductivity, and abrasive resistant capacity		2018 [86]
GO/SiO_2_	NR	Spray sputtering drying method, dry-ice expansion pre-dispersion method, and mechanical stirring method	Tensile strength, tear strength, and wear resistance		2018 [69]
mGO/SiO_2_	SBR	Preparing the mGO firstly; mixing the latex with silica secondly; mechanically mixing with other additives in Banbury mixer finally	Dry and wet braking and rolling resistance	Green tires	2018 [90]
mGO/mSiO_2_	SBR	Preparing the mGO, followed by latex mixing with modified silica and SBR	Mechanical properties and thermal conductivity	Tire Products	2019 [91]
mGO/mSiO_2_	NR	Chemical reaction method was used to prepare mGO/mSiO_2_, followed by latex mixing with NR	Wear resistance, wet-skid resistance, and decreases the rolling resistance	Green tire-tread	2019 [80]
rGO/SiO_2_	SR	Sol–gel method was used to prepare rGO/SiO_2_ hybrid fillers, followed by solution mixing with SR	Thermal stability, mechanical properties, and thermal conductivity		2019 [66]
mGO/mSiO_2_	NR	Preparing the H-bonded mSiO_2_/mGO hybrid fillers, followed by latex mixing with NR	High-grip performance, superior rolling, and wear resistance	Tire-tread	2019 [80]
GO/SiO_2_	SBR	Preparing the SiO_2_-GO hybrid nanofillers by in situ hydrolysis and condensation of tetraethyl orthosilicate on the surface of GO nanosheets, followed by latex mixing with SBR	Mechanical property and thermal conductivity		2019 [70]
GO/SiO_2_	Devulcanized NR/SBR	Mechanical mixing	Tensile strength, higher storage modulus, and lower tan δ		2019 [67]
GO/SiO_2_	SR	The SiO_2_ nanoparticles were coated on the surface of GO by sol–gel method, followed by solution mixing with PDMS to obtain the composites	Thermal stability, mechanical properties, and thermal conductivity		2019 [66]
rGO/SiO_2_	NR	Wet compounding and latex mixing process using a novel interface modifier cystamine dihydrochloride	Mechanical property and low water vapor permeability		2020 [65]
GO/SiO_2_	SBR	Mechanical mixing	Mechanical property, lowest crack growth rate, and longer fatigue lives		2020 [92]
GO/mSiO_2_	NR	Bridging GO and nanosilica by covalent bonding, followed by latex mixing with NR	Tensile strength, covalent bridging, and orientations of chain segments in interfacial regions		2020 [85]
GO/SiO_2_	NR	Preparing the SiO2@GO using the sol–gel method, followed by mechanical mixing with NR	Crosslink Density and storage modulus in all temperature range	compressive or abrasive applications	2020 [64]
GNs/SiO_2_	NR	Preparing the GNs/NR masterbatch by latex mixing, followed by mechanical mixing with silica and rubber	Thermal conductivity and antistatic property		2021 [63]
GO/mSiO_2_	NR	Preparing the mSiO_2_-KH550/GO hybrid fillers by solution mixing and freeze-drying process, followed by mixing with NR by latex co-precipitation method	Mechanical properties, low heat generation performance, and thermal conductivity	Tire rubber	2022 [84]
GE/Paraffin@SiO_2_	SR	Mechanical mixing	Thermal conductivity	Passive thermal regulation	2022 [89]
mGO/SiO_2_	SSBR	Preparing the mGO/SSBR masterbatch by latex mixing, followed by mechanical mixing with silica and rubber	Rolling resistance, wet skid, and abrasion resistances	green tire	2023 [73]
GNPs/paraffin@SiO_2_	SR	Solution mixing and met blending	Mechanical property, thermal conductivity, and low leakage rate	Thermal storage	2023 [88]
rGO/NSi	NR	Mechanical mixing	Decrease the heat build-up and Akron abrasion loss, increase the mechanical property	Green tire	2023 [93]

SSBR: solution styrene-butadiene rubber; mSiO_2_: modified silica; GNs: graphene nanosheets; NSi: silicon dioxide nanoparticles.

**Table 3:** Summary of graphene (graphene derivatives)/carbon nanotube composites.

Hybrid Fillers	Rubber Matrix	Preparation Methods	Improved Properties	Application	Year and Reference
GE/MWNTs	SR	Solution mixing	Electrical property		2012 [109]
GE/MWCNTs	PDMS	Using organic sol–gel chemistry method to prepare GE/MWCNT aerogel, followed by backfilling PDMS to obtain the composites	Mechanical property and electrical conductivity		2013 [118]
GE/MWCNTs	SR	Solution mixing	Tensile strength, Young’s modulus, crosslink density, thermal stability, and solvent resistance		2014 [112]
rGO/CNT	PDMS	CNTs were in situ formed on a reduced graphene sheet by the catalytic growth procedure, followed by solution mixing with PDMS to obtain the composites	Lower interfacial contact electrical resistance and absorbing EM power		2014 [113]
GE/CNTs	NR	Latex mixing	Fracture toughness and tensile strength, also suppresses the crack growth		2015 [100]
GNPs/MWCNTs	EPDM	Mechanical mixing	Electrical conductivity, tensile strength, Young’s modulus, and tear strength		2015 [101]
GE/CNTs	PDMS	Mechanical mixing	Cyclic adhesion and electrical conductivity	Conductive dry adhesives	2016 [116]
GE/CNTs	PDMS	Solution mixing	Electrical conductivity	Stretchable conductors	2016 [111]
GF/CNTs	PDMS	PDMS and CNTs solutions were deposited onto the surface of GF/Ni foam, followed by etching the Ni skeleton to obtain the composites	Electrical conductivity and EMI SE		2017 [119]
GNPs/CNTs	SR	Simultaneous reactions of beer’s yeast fermentation and the gelation of liquid SR mixed with GNPs and CNTs	Stretchability, and damage resistance with reduced density	Bionic composites	2017 [98]
rGO/MWCNTs	NBR	Mechanical mixing	Mechanical strength, electrical conductivity, and decreased ice adhesion strength		2018 [97]
GE/CNTs	NBR	Mechanical mixing	Icephobic surface	Components that need to survive in extreme cold environments	2019 [99]
rGO/CNT	SBR	Preparing the rGO-CNT hybrids firstly; mixing the latex with SBR secondly; using freeze-drying to obtain the composite foam finally	Electrical conductivity and stretchable performance		2019 [102]
GE/CNT	SR	Solution mixing	Sensing property		2019 [105]
GE/CNTs	SR	Solution mixing	Electrical conductivity, optical transmittance, and mechanical adhesion		2019 [96]
rGO/MWCNT	CR	Solution mixing to prepare the hybrid fillers, followed by mechanical mixing with CR	Thermal stability, dielectric conductivity, and high energy storage efficiency	Electronic devices	2020 [117]
rGO/ MWCNTs	PDMS	Using the freeze-casting method to prepare 3D rGO/MWCNTs foam, followed by impregnating liquid PDMS into the hybrid foam	Thermal conductivity compressive strength and electromagnetic interference shielding effectiveness	Carbon-based electromagnetic shielding materials	2020 [120]
GE/CNTs	SR	Solution mixing	Reduced the percolation threshold and enhancing strain sensing		2020 [103]
rGO/CNT	NR	Latex mixing	Electrical conductivity and mechanical property		2020 [104]
rGO/ MWCNTs	SR	Preparing the 3D rGO/MWCNTs aerogel, followed by solution mixing with SR	Thermal conductivity, electric conductivity, and EMI SE	Flexible electronic devices	2021 [28]
GO/MWCNTs	IIR	Latex mixing with ice expansion and pre-dispersion flocculation method	Thermal conductivity		2022 [114]
GO/CNT	Waste silicone rubber	Solution mixing followed by freeze-drying	Mechanical properties, electrical and thermal conductivity, and detection range	Wearable sensors and electronic skin	2022 [121]
GE/PNTs	EPDM	Mechanical mixing	Ablation performance and mechanical property	High performance ablative materials	2022 [106]
GE/CNTs	NBR	Emulsification and mechanical blending	Wear performance		2022 [107]
rGO/CNT	NR	Solution and latex mixing	Strain sensing properties	Wearable electronic device	2022 [108]

MWCNTs: multi-wall carbon nanotubes; CNTs: Carbon nanotubes; CR: chloroprene rubber; PNTs: polyphosphazene nanotubes; EMI SE: electromagnetic interference shielding effectiveness.

**Table 4:** Summary of graphene (graphene derivatives)/metal oxide composites.

Hybrid Fillers	Rubber Matrix	Preparation Methods	Improved Properties	Application	Year and Reference
GE/NZG	NR	Using the solvothermal method to prepare the GE/NZG hybrid fillers, followed by mechanical mixing with NR	Mechanical performance and gas barrier properties		2015 [139]
GE/ZnO	NR	Preparing ZnO–GE using the sol–gel method, followed by mechanical mixing with NR	Mechanical property, wet-grip property, and rolling resistance		2015 [138]
GO/Fe_3_O_4_	NR	Latex mixing	Corrosion resistance and flexible anticorrosive properties		2017 [132]
rGO/Fe_3_O_4_	NR	Latex mixing	Magnetic property and EMI SE		2018 [26]
GnPs/S-Al_2_O_3_	SR	Solution mixing	Thermal conductivity, tensile strength, and low density	Heat dissipation of light-emitting diode	2018 [141]
GO/ZnO	CR	Preparing the GO/ZnO by electrostatic adsorption and the in situ growth method, followed by mechanical mixing with CR to obtain the composites	Crosslinking efficiency and density, tensile modulus at 300% elongation, and tensile strength		2019 [137]
GE/CeO_2_	PMVQ	Solution mixing	Thermal stability and mechanical properties		2019 [144]
rGO/Al_2_O_3_	NR	Preparing rGO/Al_2_O_3_ using the electrostatic self-assembly method, followed by latex mixing with NR	Thermal conductivity		2020 [142]
rGO/mAl_2_O_3_	NR	Solution mixing	Thermal conductivity, mechanical properties, and electrical insulation	Electronic packaging materials	2021 [143]
FLG/Fe_3_O_4_, FLG/TiO_2_	SR	Solution mixing	Mechanical, actuation, and magnetic properties	Magnetic sensor	2021 [145]
rGO/Fe_3_O_4_	SR	Preparing the rGO@Fe_3_O_4_ nanocomposites using the thermal decomposition method, followed by mechanical mixing with SR	High microwave-absorption efficiency and wide absorption bandwidth.	Aerospace and flexible electronics	2021 [133]
GON/Fe_3_O_4_	PVDF-SBR	Preparing the PVDF-SBR/GON and PVDF-SBR/Fe_3_O_4_ masterbatch using the solution mixing method, followed by mechanical mixing with two masterbatches and PVDF-SBR to obtain the composites	Thermal, mechanical, rheological, electrical, and magnetic properties		2021 [135]
rGO/Fe_3_O_4_	NR	Latex mixing	EMI-shielding stability under tensile permanent deformation, cyclic stretching, and cyclic bending; sensing properties	Wearable electronic flexibility devices	2021 [27]
rGO/Fe_3_O_4_	SR	Using hydrothermal and freeze-drying processes to prepare rGO/Fe_3_O_4_ hybrid fillers, followed by solution mixing with SR	Microwave-absorbing properties		2022 [146]
MLG/Fe_3_O_4_	NR	Solvent mixing	Mechanical and magnetic properties		2022 [134]
rGO/Fe_3_O_4_	ENR	In situ reduction with latex mixing	Mechanical, electrical conductivity, magnetic, and sensing properties		2022 [147]
GE/ZnO	XNBR	Mixing GE and XNBR latex in a ball-mill tank, followed by mechanical mixing with ZnO	Electrical conductivity, mechanical property, and microwave absorption		2022 [140]
GE/Al_2_O_3_	SR	Mechanical mixing	Nonlinear electrical conductivity, thermal conductivity	Cable accessory	2023 [148]

GON: graphene oxide nanosheets; MLG: multilayer graphene; PVDF: polyvinylidene difluoride; NZG: ZnO nanoparticles; FLG: few layers graphene; S-Al_2_O_3_: spherical alumina; PMVQ: phenyl silicone rubber composites; XNBR: carboxylated butadiene acryloni-trile; mAl_2_O_3_: modified alumina oxide.

**Table 5:** Summary of graphene (graphene derivatives)/nanowires (microwire, nanofiber) composites.

Hybrid Fillers	Rubber Matrix	Preparation Methods	Improved Properties	Application	Year and Reference
rGO/CNC	NR	Preparing the rGO/CNC hybrid fillers by solution mixing, followed by latex mixing with NR	Electric conductivity and mechanical properties	Sensing materials with superior resistivity responses for organic liquids	2016 [164]
GA/AGNWs	PDMS	Preparing the GA using the hydrothermal reduction and freeze-drying process, followed by solution mixing with AgNWs and PDMS to obtain the composites	Electrical conductivity	Skin patch sensors and wearable electronic devices	2017 [149]
rGO/AgNWs	PDMS	Preparing the rGO/AgNWs hybrid fillers using the sol–gel method with the freeze-drying process, followed by backfilling PDMS into the hybrid filer to obtain the composites	EMWs absorption, good compression, excellent thermal stability, and flame-retardant properties		2019 [150]
GE/CNF	NR	Using solution mixing to prepare the hybrid fillers, followed by latex mixing with NR to obtain the composites	Mechanical property and electrical conductivity		2019 [162]
GNPs/cobalt nanowire	NR	Mechanical mixing	Tensile strength, tear strength, microwave shielding ability, and magnetic property		2020 [156]
rGO/SiCNWs	SR	Solution mixing to prepare hybrid fillers, ice-templated assembly of hybrid fillers, and then infiltrated with the SR to obtain the composites	Thermal conductivity		2020 [155]
Graphene fibers/magnetic microwires	SR	Solution mixing	EMI SE and microwave reflectivity		2020 [161]
rGO/CNF	NBR	Pickering emulsion and the hot-pressing method	EMI SE and EMI SE stability	Piezoresistive Sensor	2021 [154]
GE/ANFs	NR	Using the solution mixing method to prepare the GE/ANF hybrid fillers, followed by mechanical mixing with NR	Enhanced mechanical interlocking and stress transfer between fiber and rubber		2021 [163]
rGO aerogels/Cellulose carbon aerogel	PDMS	Preparing cellulose carbon aerogel @rGO aerogels by vacuum impregnation, freeze-drying, and thermal annealing, followed by backfilling PDMS to obtain the composites	EMI SE, thermal stability, and thermal conductivity		2021 [159]
Functionalized GO/ANF	SBR	Using the solution mixing method to prepare functionalized GO/ANF hybrid fillers, followed by mechanical mixing with SBR	Tensile strength, elongation at break, abrasion resistance, and rolling resistance	Tire tread	2022 [157]
GE/CGF	NBR	Mechanical mixing	Elongation at break and dielectric property		2022 [165]
rGO/ANFs	NR	Latex mixing	Strain sensing properties	Wearable electronic device	2022 [160]
rGO/CNC hybrid aerogel	EPDM	Preparing the rGO/CNC hybrid aerogel, followed by solution mixing with EPDM	Oil/water separation performance and EMI SE		2023 [166]

GA: graphene aerogel; CNC: cellulose nanocrystals; AgNWs: silver nanowires; ANFs: aramid fibers; SiCNWs: silicon carbide nanowires; CNF: cellulose nanofibrils; CGF: chopped glass fiber; EMWS: electromagnetic waves.

**Table 6:** The summary of other graphene (graphene derivatives) hybrid filler composites.

Hybrid Fillers	Rubber Matrix	Preparation Methods	Improved Properties	Application	Year and Reference
GS/FeCo	NR/SiC	Using the wet impregnation and thermal reduction reaction method to prepare GS/FeCo hybrid fillers, followed by mechanical mixing with NR to obtain the composites	Reflection loss		2018 [167]
GO/ND	XSBR	Attaching ND particles to GO in solution mixing by using diisocyanate as the coupling agent, followed by latex mixing with XSBR	Tensile strength, thermal stability, and the thermos-physical properties		2018 [178]
GE/ZDMA	EPDM	Mechanical mixing	Tensile strength, elongation at break, total crosslinking density, and sealing resilience		2019 [169]
rGO/BN	NR	Preparing the three-dimensional rGO/BN hybrid fillers by using the surface modification and ice-templated methods, followed by infiltrating the NR into the filler to obtain the composites	Thermal conductivity and mechanical property	Electronic packing materials	2019 [174]
rGO/BN	NR	GO-assisted gelation process and latex mixing	In-plane thermal conductivity, flame-retardant ability, and antistatic performance		2020 [173]
rPGO/MoS_2_	NR	Preparing the hybrid fillers by solution mixing, followed by latex mixing with NR	Mechanical properties, dielectric properties, and thermal stability		2021 [183]
GNPs/Pt	SR	Solution mixing	Polymer electrolyte membrane fuel cell performance	Polymer electrolyte membrane fuel cel	2021 [176]
GO/NC	NR	Latex mixing	High water vapor permeability, tensile strength, and breaking elongation	Latex medical gloves	2021 [177]
GNP/EIP	SR	Solution mixing	Mechanical property and magnetic effect		2022 [184]
GE/T-ZnOw	PLA/NR	Melt blending	Thermal conductivity, high recovery rate, and thermal stability	Electronic products	2022 [185]
SGO/Pd	SR	Solution mixing	Hydrogen elimination	New getter with environmental adaptability	2022 [171]
CGF/GE	NBR	Mechanical mixing	Elongation at break and dielectric property		2022 [165]
GO/COL	SBR	Solution mixing	Mechanical and biodegradable properties	Green Tire	2022 [181]
Graphene-coated iron	NR	Preparing graphene-coated iron nanoparticles, firstly, followed by mechanical mixing with NR	Thermal oxygen aging performance	Magnetorheolog-cal elastomers	2022 [172]
GE/SiC whisker	SR	Solution mixing	Thermal conductivity	Thermal interface ma- terial for LED package	2022 [186]
SiC/GO	NR	Preparing the SiC/GO/NR masterbatch by latex mixing, followed by mechanical mixing with NR	Tensile strength, thermal conductivity, and low heat build-up		2022 [187]
rGO/BN	NR	Latex mixing with freeze-drying	Tensile strength and thermal conductivity		2022 [175]
GO/Lingin	SBR	Solution and latex mixing	Stable heat tolerance behavior, mechanical, and biodegradable properties	Green tire	2022 [179]
GNPS/BT	FSR	Mechanical mixing	Dielectric property	Dielectric elastomer	2022 [170]
GO/fibrous sepiolite	NR	Latex mixing	Tensile strength and elongation at break		2022 [180]
mGO/Ag	NR	Preparing the Ag/PDA-GO hybrid fillers, followed by latex mixing with NR	Tensile strength, tear strength and modulus at 100% and 300% strains, antibacterial, and barrier properties		2022 [168]
GO/MMA	NBR	Mechanical mixing	Dispersion stability		2023 [188]
GO/CI	SR	Preparing the GO-CI hybrid fillers by γ-ray irradiation, followed by solution mixing with SR	γ-ray radiation shielding ability, thermal conduction, and mechanical recovery properties	Radiation hardening material	2023 [191]

ZDMA: Zinc dimethacrylate; NC: zwitterionic chitin nanocrystals; ND: nanodiamond; BN: boron nitride; EIP: belectrolyte-iron particles; T-ZnOw: tetrapod zinc oxide whisker; PLA: polylactic; COL: collagen, BT: barium titanate; MMA: methyl methacrylate; FSR: fluorosilicone rubber; XSBR: carboxylated styrene-butadiene rubber; GS: graphene sheets; rPGO: porous reduced graphene oxide; SGO: silanized graphene oxide; CI: carbonyl iron.

**Table 7:** Summary of three-phase graphene three-phase/hybrid filler rubber composites.

Hybrid Fillers	Rubber Matrix	Preparation Methods	Improved Properties	Application	Year and Reference
GO/CNT/CB	NR	Latex mixing followed by mechanical mixing	Improving the fatigue crack growth resistance and reduced the heat build-up		2018 [193]
GE/CB/SiO_2_	NR/SBR	Mechanical mixing with a planetary ball mill machine	Wear resistance		2018 [195]
MLG/ATH/CB	HNBR	Preparing the masterbatch by ultrasonically assisted solution mixing, followed by mechanical mixing	Mechanical properties and flame retardancy		2018 [203]
GE/CB/SiO_2_	NR	Mechanical mixing	Mechanical property		2020 [196]
mGO/ND/nanoZnO	NR	Preparing the hybrid fillers by solution mixing and freeze-dried process, followed by latex mixing with NR	Mechanical property and vulcanization characteristics		2020 [199]
GE/SiO_2_/TiO_2_	EPDM/SR	Mechanical mixing	Thermal stability and thermal conductivity		2020 [202]
rGO/TiO_2_/Ni	NR	Mechanical mixing	Tensile strength, Young’s modulus, elongation, and thermal stability		2020 [200]
GNPs/CB/SiO_2_	SBR/BR	Mechanical mixing	Mechanical property		2022 [197]
HS@SiO_2_/GO/CB	NBR	Preparing the GO/silica hybrid fillers which anchored HS@SiO_2_ on the GO nanosheet, followed by latex mixing with NBR and CB to obtain the HS@SiO_2_/GO/CB/NBR composites.	Crosslink density and tensile strength		2022 [198]
rGO/Hf-SiO_2_/CNTs	Elastic rubber band	Solution mixing	Sensing property		2022 [194]
GE/CNTs/CNF	SR	Solution mixing	Electrical conductivity, low conductive percolation threshold, and excellent piezoresistivity		2022 [206]
ATH/MGFs/GNPs	SR	Solution mixing	Fire retardancy, mechanical strength, and hydrophobicity whilst		2022 [204]
rGO/CNTs/SiO_2_	HNBR	Preparing RGO-CNTs hybrid fillers using the electrostatic assembly method firstly; growing SiO_2_ in situ on the RGO-CNTs to obtain the rGO/CNTs/SiO_2_ hybrid fillers Secondly, mechanically mixing with HNBR to obtain the composites finally	Static and dynamic mechanical properties		2022 [192]
GE/CB/SiO_2_	SBR/BR	Mechanical mixing	Mechanical property		2022 [197]
GO/CMCS/Ag	NR	Latex-dipping	Mechanical property, barrier property, and antiracial property application	Medical gloves	2023 [207]
MWCNT s/GE/ /Fe_3_O_4_	SR	Solution mixing followed by 3D printing	Stretchability, sensitivity, response/recovery times, and durability	Sign language pose recognition, non-contact sensing, human-computer interaction and electronic skin	2023 [205]
rGO/MAH/SiO_2_	SBR	Preparing the hybrid fillers SBR masterbatch using the latex mixing, followed by mechanical mixing with rubber matrix	300% modulus, tensile strength and abrasion resistance, lower heat build-up		2023 [208]
GE/CNT/CF	SR	Solution mixing	Electrical conductivity and heat resistance		2023 [209]

HNBR: hydrogenated acrylonitrile-butadiene rubber; BR: butadiene rubber; MGFs: milled glass fires; Hf-SiO_2_: hydrophobic fumed silica; HS@SiO_2_: ultra-small mercaptopropyl doped silica; ATH: aluminum trihydroxide; CNF: cellulose nanofibrils; MGFs: milled glass fibers; CMSs: carboxymethyl chitosan; MAH: maleic anhydride; CF: carbon fiber.
